# Mitochondrial Regulation of CD8⁺ T Cells: Mechanisms and Therapeutic Modulation

**DOI:** 10.1002/advs.202503095

**Published:** 2025-06-23

**Authors:** Xu Chen, Pei Lin, Ye Lu, Jiarong Zheng, Yunfan Lin, Zihao Zhou, Li Cui, Xinyuan Zhao

**Affiliations:** ^1^ Stomatological Hospital School of Stomatology Southern Medical University Guangzhou Guangdong 510280 China; ^2^ Department of Dentistry The First Affiliated Hospital Sun Yat‐Sen University Guangzhou 510080 China; ^3^ School of Dentistry University of California Los Angeles CA 90095 USA

**Keywords:** CD8⁺ T cells, immunotherapy, metabolic reprogramming, mitochondrial dynamics, T cell exhaustion

## Abstract

Mitochondria are integral to the regulation of CD8^+^ T cell function, critically influencing processes such as activation, differentiation, and long‐term persistence during immune responses. Emerging evidence highlights the detrimental impact of mitochondrial dysfunction on CD8^+^ T cell activity, contributing to immune exhaustion and impairing both antitumor and antiviral immunity. This underscores the importance of understanding and modulating mitochondrial dynamics to optimize T cell‐based immunotherapies. In this review, a comprehensive and in‐depth analysis of the essential mitochondrial processes—including biogenesis, redox homeostasis, and metabolic reprogramming is provided—that govern CD8^+^ T cell function and are intricately linked to their therapeutic potential. The current strategies aimed at enhancing mitochondrial function in CD8^+^ T cells are also examined, focusing on both metabolic reprogramming and mitochondrial‐targeted interventions. Despite these promising approaches, several significant challenges remain, such as achieving selective targeting, addressing mitochondrial plasticity, and mitigating off‐target effects. Overcoming these obstacles will be crucial to improving the clinical efficacy and safety of mitochondrial modulation therapies. As the understanding of mitochondrial dynamics within CD8^+^ T cells continues to evolve, there is growing potential to leverage these insights to improve immune‐based therapies across a range of diseases, including cancer and viral infections.

## Graphical abstract

This article comprehensively explores the pivotal role of mitochondrial function in CD8⁺ T cell immunotherapy. It highlights how mitochondrial dynamics regulate T cell activation, differentiation, and persistence, and how mitochondrial dysfunction leads to immune exhaustion and impaired antitumor/antiviral responses. The review also discusses strategies to enhance CD8⁺ T cell function through metabolic reprogramming and mitochondrial‐targeted interventions, offering insights for developing novel immunotherapies.

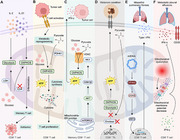

## 1 Introduction

CD8^+^ T cells are critical mediators of immune responses, playing a pivotal role in the defense against infections, the surveillance of tumorigenesis, and the regulation of autoimmune diseases.^[^ ^1^, ^2^ ^]^ These cytotoxic lymphocytes exert their functions through the recognition and elimination of infected or malignant cells, primarily by inducing apoptosis via the release of cytotoxic molecules such as perforin and granzymes^[^ ^3^ ^]^ The effectiveness of CD8^+^ T cells in these processes is largely dependent on their metabolic flexibility and mitochondrial function. Mitochondria, as the powerhouse of the cell, are crucial for sustaining the high energy demands of T cell activation, differentiation, and effector functions.^[^ ^4^ ^]^ They govern ATP production via oxidative phosphorylation (OXPHOS), support the synthesis of critical metabolites, and regulate calcium signaling and apoptosis, all of which are essential for maintaining CD8^+^ T cell activity and longevity.^[^ ^5^ ^]^

However, the integrity of mitochondrial function in CD8^+^ T cells can be compromised under various pathophysiological conditions, leading to immune dysfunction and disease progression. In chronic infections, cancers, and autoimmune diseases, mitochondrial abnormalities are often observed, including reduced mitochondrial biogenesis, impaired OXPHOS, and increased mitochondrial reactive oxygen species (ROS) production.^[^ ^6^ ^]^ These defects contribute to the exhaustion and dysfunction of CD8^+^ T cells, impairing their ability to mount effective immune responses. For instance, PD‐1 high HBV‐specific CD8^+^ T cells exhibit metabolic inflexibility, relying on glucose due to mitochondrial dysfunction. These HBV‐specific T cells display enlarged mitochondria with reduced potential, limiting their ability to switch metabolic pathways.^[^ ^7^ ^]^ In addition, CD28 costimulation enhances the activation, function, and proliferation of CD8⁺ TILs in renal cell carcinoma by reprogramming their metabolism. This process increases glycolysis and mitochondrial oxidative metabolism, leading to greater mitochondrial fusion, membrane potential, and mass, highlighting the critical role of mitochondrial metabolism in restoring CD8⁺ TIL efficacy in the tumor microenvironment (TME).^[^ ^8^ ^]^

In this review, we systematically and critically examine the key aspects of mitochondrial function—such as biogenesis, redox balance, and metabolism—that are closely linked to enhancing CD8^+^ T cell‐based immunotherapy. In addition, a comprehensive overview of current strategies aimed at improving mitochondrial function in CD8^+^ T cells is also provided. Moreover, the challenges in modulating mitochondrial function for therapeutic purposes are discussed, along with future directions for overcoming these obstacles. By analyzing the role of mitochondria in CD8^+^ T cell immunotherapy, this review highlights novel therapeutic opportunities and explores the potential for mitochondrial‐based interventions to improve immune responses and restore immune homeostasis across various disease settings.

## 2 The Crucial Role of Mitochondria in Mediating CD8^+^ T Function

**Figure 1:**
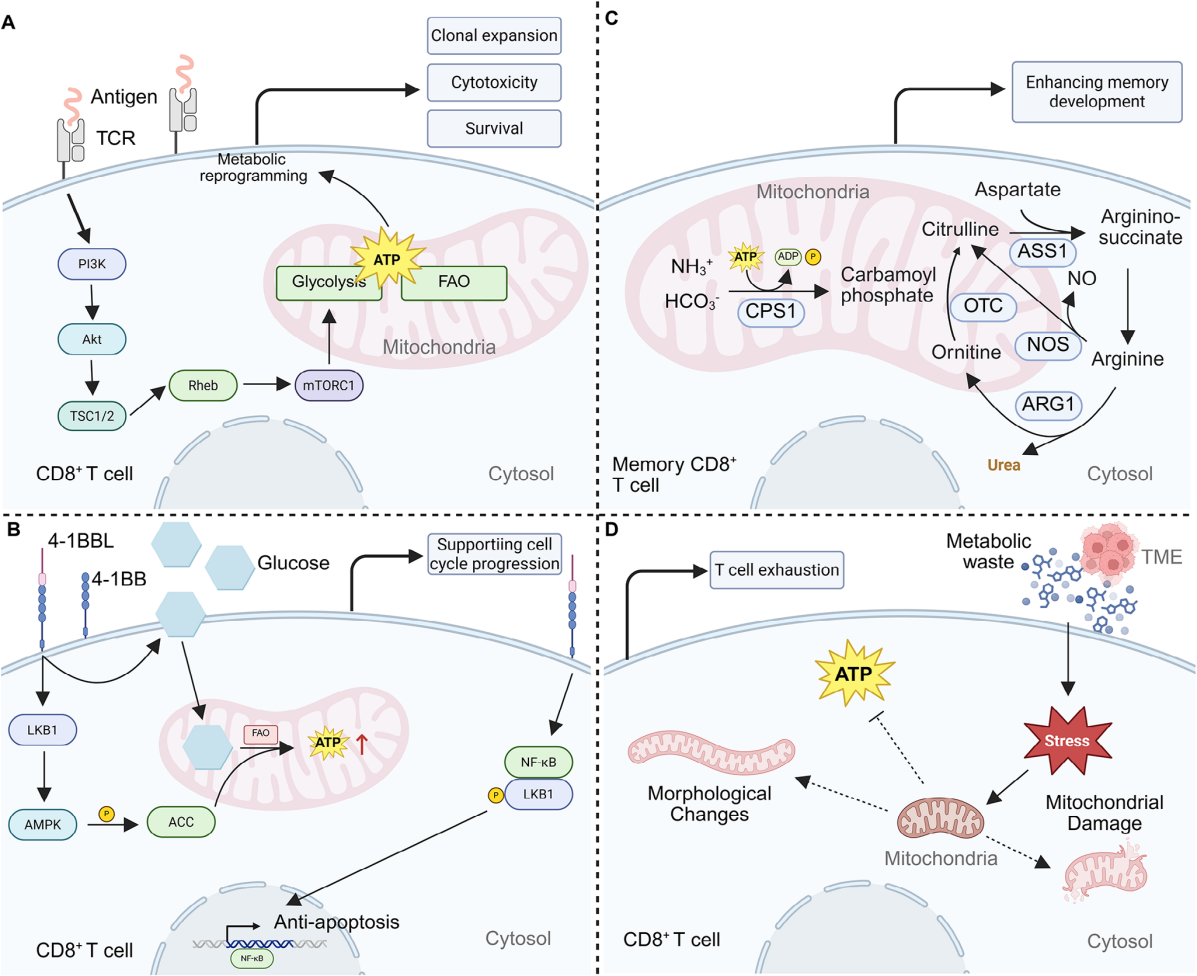
The crucial role of mitochondria in mediating CD8^+^ T function. A) TCR engagement induces PI3K‐Akt signaling, leading to mTORC1 activation via TSC1/2 and Rheb, thereby promoting metabolic reprogramming. Enhanced glycolysis and FAO support ATP production, driving clonal expansion, cytotoxicity, and survival of activated CD8⁺ T cells. B) 4‐1BB co‐stimulation activates LKB1‐AMPK signaling, facilitating FAO and ATP generation. Concurrently, NF‐κB activation promotes anti‐apoptotic pathways, while glucose metabolism supports cell cycle progression, ensuring sustained CD8⁺ T cell function. C) Memory CD8⁺ T cells rely on mitochondrial metabolism and the urea cycle, where CPS1, OTC, ASS1, ARG1, and NOS coordinate nitrogen metabolism. The conversion of aspartate to argininosuccinate supports NO production, contributing to enhanced memory development and longevity. D) Metabolic stress in the TME induces mitochondrial dysfunction in CD8⁺ T cells, leading to reduced mitochondrial content, altered morphology, and impaired oxidative metabolism, contributing to T cell exhaustion and dysfunction.

Mitochondria are indispensable for the function, survival, and differentiation of CD8^+^ T cells, acting as key regulators of cellular metabolism and signaling.^[^ ^9^, ^10^ ^]^ Upon antigen recognition, CD8^+^ T cells undergo a metabolic reprogramming process that is crucial for fulfilling the increased energy and biosynthesis demands associated with clonal expansion, cytotoxicity, and cytokine production. These metabolic shifts involve a coordinated activation of mitochondrial pathways, including OXPHOS, fatty acid oxidation (FAO), and glycolysis, which provide the ATP and biosynthetic intermediates required for cellular processes such as protein synthesis, membrane remodeling, and cell division (**Figure** **1**A).^[^ ^11^ ^]^ Notably, mitochondria also play a central role in maintaining cellular redox balance and modulating oxidative stress responses, thereby influencing T cell survival and functionality during immune responses.^[^ ^2^, ^12^ ^]^

In the context of T cell activation and differentiation, mitochondria function not only as energy providers but also as signaling hubs. For instance, 4‐1BB signaling enhances CD8⁺ T cell proliferation by activating glucose and fatty acid metabolism, crucial for meeting energy and biomass demands. This process involves the LKB1‐AMPK‐ACC pathway, with fatty acid metabolism driving cell cycle progression and anti‐apoptotic effects. Inhibition of FAO, but not glycolysis, blocks these 4‐1BB‐induced effects, underscoring the critical role of mitochondrial metabolism in supporting T‐cell function and survival (Figure 1B).^[^ ^13^ ^]^ Mitochondria are also critical in the development and maintenance of memory CD8^+^ T cells. CD8⁺ memory T cells employ the carbamoyl phosphate metabolic pathway for ammonia clearance, enhancing memory development. This involves β‐hydroxybutyrylation upregulating carbamoyl phosphate synthetase 1, facilitating arginine synthesis and its mitochondrial processing into urea and ornithine, alongside nitric oxide and citrulline production (Figure 1C).^[^ ^14^ ^]^

Mitochondrial dysfunction or impaired metabolic reprogramming can severely compromise CD8^+^ T cell effector function, survival, and persistence. In the TME, T cells often experience metabolic stress due to nutrient deprivation and the accumulation of metabolic waste products. This environment induces mitochondrial dysfunction, leading to a decrease in mitochondrial content, altered mitochondrial morphology, and impaired oxidative metabolism, which can contribute to T‐cell exhaustion (Figure 1D).^[^ ^15^ ^]^ For instance, glutamine deprivation in the TME impairs mitochondrial function in infiltrating CD8⁺ T cells in hepatocellular carcinoma, leading to increased apoptosis and dysfunction. This metabolic challenge suppresses the T cells' ability to secrete cytotoxic molecules like perforin and granzyme B, exacerbating their dysfunction and highlighting the pivotal role of glutamine in sustaining mitochondrial health and T cell efficacy in cancer.^[^ ^16^ ^]^

## 3 Implications of Abnormal Mitochondrial Function in CD8^**+**^ T Cells in Immune‐Related Diseases

**Figure 2:**
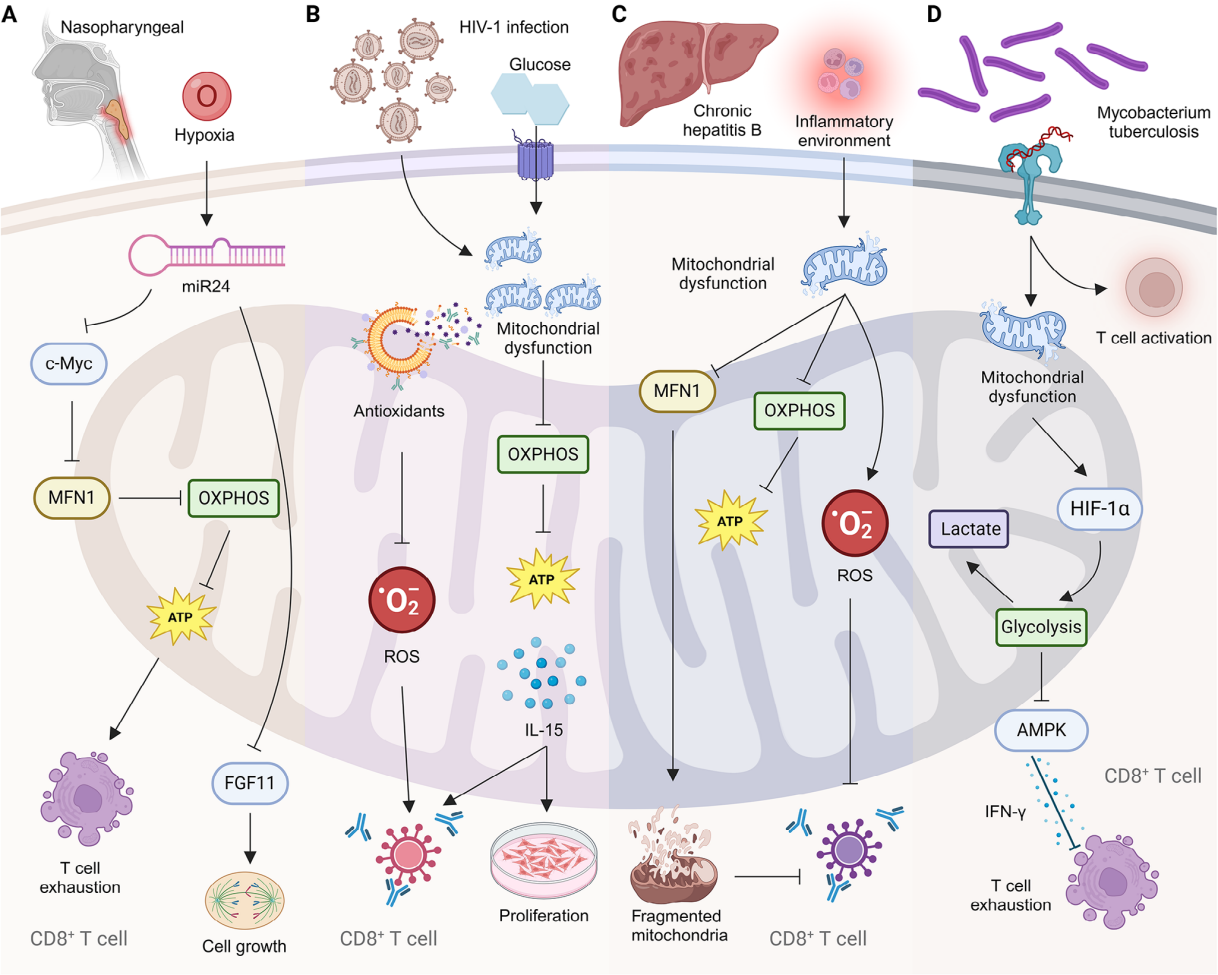
Mitochondrial dysfunction in CD8⁺ T cells under pathological conditions. A) Hypoxia in the nasopharyngeal microenvironment induces miR‐24, leading to c‐Myc suppression and reduced MFN1 expression, thereby impairing OXPHOS and ATP production. This metabolic dysregulation promotes CD8⁺ T cell exhaustion, while FGF11 activation partially supports cell growth. B) HIV‐1 infection disrupts mitochondrial function, exacerbated by glucose metabolism imbalance, leading to excessive ROS accumulation. Antioxidant mechanisms attempt to mitigate oxidative stress; however, mitochondrial dysfunction limits OXPHOS‐dependent ATP synthesis, impairing IL‐15‐mediated CD8⁺ T cell proliferation and leading to mitochondrial fragmentation. C) Chronic hepatitis B and inflammatory conditions drive mitochondrial dysfunction by disrupting MFN1 and OXPHOS, resulting in elevated ROS production. This oxidative stress contributes to mitochondrial fragmentation and restricts CD8⁺ T cell expansion. D) Mycobacterium tuberculosis infection induces T cell activation, yet sustained mitochondrial dysfunction promotes a metabolic shift toward glycolysis, driven by HIF‐1α. This metabolic reprogramming leads to lactate accumulation and AMPK activation, ultimately impairing IFN‐γ production and promoting CD8⁺ T cell exhaustion.

Abnormal mitochondrial function in CD8^+^ T cells is closely linked to the development of various immune‐related diseases, given the critical role these cells play in immune responses. CD8^+^ T cells are essential for the elimination of infected or cancerous cells, as well as the regulation of autoimmune responses.^[^ ^2^ ^]^ When mitochondrial function in CD8^+^ T cells is disrupted, it can impair their activation, differentiation, and effector functions, contributing to the onset and progression of numerous immune‐related disorders.^[^ ^17^, ^18^ ^]^ For instance, hypoxia in the TME drives CD8^+^ T cell exhaustion by inducing mitochondrial fragmentation and reduced ATP production, linked to the downregulation of MFN1 and upregulation of miR‐24. Targeting miR‐24 to restore mitochondrial function may enhance immunotherapy efficacy in nasopharyngeal carcinoma (**Figure** **2**A).^[^ ^19^ ^]^ In addition, in HIV‐1 infection, CD8^+^ T cells exhibit an exhaustion signature characterized by metabolic dysregulation, including increased Glut‐1 expression and impaired mitochondrial function. This phenotype persists during antiretroviral therapy but improves with interventions targeting mitochondrial health, such as antioxidants, mitochondrial dynamic modulators, and IL‐15 therapy. These findings underscore mitochondria as therapeutic targets for rejuvenating antiviral CD8^+^ T cell responses in HIV‐1 infected individuals (Figure 2B).^[^ ^20^ ^]^ Similarly, exhausted HBV‐specific CD8^+^ T cells in chronic hepatitis B show profound mitochondrial dysfunction, impairing their antiviral activity. Mitochondria‐targeted antioxidants can restore mitochondrial function and improve CD8^+^ T cell responses, underscoring the crucial role of mitochondrial metabolism in T cell exhaustion (Figure 2C).^[^ ^21^ ^]^ Moreover, SARS‐CoV‐2 infection induces mitochondrial dysfunction in T cells, contributing to rapid lymphocytopenia and impaired functionality. Both CD4^+^ and CD8^+^ T cells show increased mitochondrial dysfunction across all patient stages, with severe cases demonstrating a lower proportion of functional mitochondria in memory CD8^+^ T cells. This dysfunction hampers T‐cell activation and responsiveness, playing a key role in the dysregulated immune response during acute infection and influencing disease severity and progression.^[^ ^22^ ^]^ Chronic Mycobacterium tuberculosis infection drives mitochondrial dysfunction in CD8^+^ T cells, leading to a shift toward glycolysis and bioenergetic quiescence, which correlates with increased inflammatory cytokine production and inhibitory receptor expression (Figure 2D).^[^ ^23^ ^]^In systemic lupus erythematosus (SLE), elevated CD38 impairs CD8^+^ T cell function by disrupting mitochondrial fitness and autophagy, thereby increasing infection risk. Targeted inhibition of CD38 in a murine lupus model restored CD8^+^ T cell effector function, enhanced mitochondrial quality via improved mitophagy, and bolstered viral clearance.^[^ ^24^ ^]^ Similarly, type I interferon exposure in SLE downregulates mitochondrial genes and impairs mitochondrial metabolism in CD8^+^ T cells, leading to enlarged mitochondria, reduced spare respiratory capacity, and increased cell death upon TCR stimulation. These effects are linked to elevated NAD^+^ consumption and impaired mitochondrial respiration, which can be rescued by NAD^+^ supplementation, highlighting a potential mechanism by which type I IFN contributes to CD8^+^ T cell dysfunction in SLE.^[^ ^25^ ^]^ In primary Sjogren's syndrome, immune cell populations in labial salivary glands exhibit distinct associations with mitochondrial pathways. Innate immune cells are linked to mitochondrial electron transport and respiratory pathways, while CD8^+^ T cells are associated with mitochondrial biogenesis and apoptosis, reflecting their unique metabolic needs. These findings underscore the crucial role of mitochondrial metabolism in regulating immune function and disease progression in autoimmune conditions.^[^ ^26^ ^]^ Although these findings consistently link mitochondrial dysfunction in CD8⁺ T cells to disease pathogenesis, much of the evidence is correlative, offering limited clarity on whether, and how, restoring mitochondrial integrity translates into sustained clinical benefit. Few studies employ prospective analyses or long‐term follow‐up to confirm that reversing mitochondrial deficits indeed ameliorate disease severity. Moreover, the functional heterogeneity of CD8⁺ T cells—ranging from naïve and memory subsets to exhausted populations—is rarely addressed with sufficient resolution, making it difficult to pinpoint which subset‐specific mitochondrial defects drive pathology. Future approaches integrating robust single‐cell metabolic profiling, in vivo functional assays, and targeted interventions will be essential for validating mitochondrial restoration as a therapeutic strategy in diverse immune‐related disorders.

## 4 Mitochondrial Regulation of CD8⁺ T Cell Fate, Metabolism, and Functional States Across Immune Contexts

Mitochondrial dysfunction in CD8^+^ T cells plays a pivotal role in driving cellular exhaustion and impairing immune responses, a characteristic feature of many immune‐related diseases. As such, targeting mitochondrial abnormalities offers a promising strategy to restore and enhance immune function.^[^ ^2^, ^9^ ^]^ For instance, 4‐1BB costimulation enhances mitochondrial capacity in CD8⁺ T cells by activating PGC1α‐mediated pathways through p38‐MAPK, improving metabolic sufficiency in the TME. This metabolic reprogramming boosts antitumor immunity, especially when combined with PD‐1 blockade.^[^ ^27^ ^]^ Similarly, MEK1/2 inhibition reprograms CD8⁺ T cells into stem cell‐like memory (T(SCM)) cells, enhancing their longevity, proliferative capacity, and multipotency by promoting mitochondrial biogenesis and FAO. This reprogramming endows T(SCM) cells with heightened recall responses and superior therapeutic efficacy in adoptive cell therapy and immune‐mediated tumor suppression, highlighting mitochondrial metabolism's pivotal role in optimizing T cell function and antitumor activity.^[^ ^28^ ^]^ Targeting mitochondrial mass, dynamics, redox balance, calcium homeostasis, and metabolism offers a promising strategy to enhance CD8^+^ T cell‐based immunotherapies, improving T cell function, preventing exhaustion, and optimizing immune responses across various diseases.

### 4.1 Mitochondrial Mass and Biogenesis in the Regulation of CD8⁺ T Cell Function

**Figure 3:**
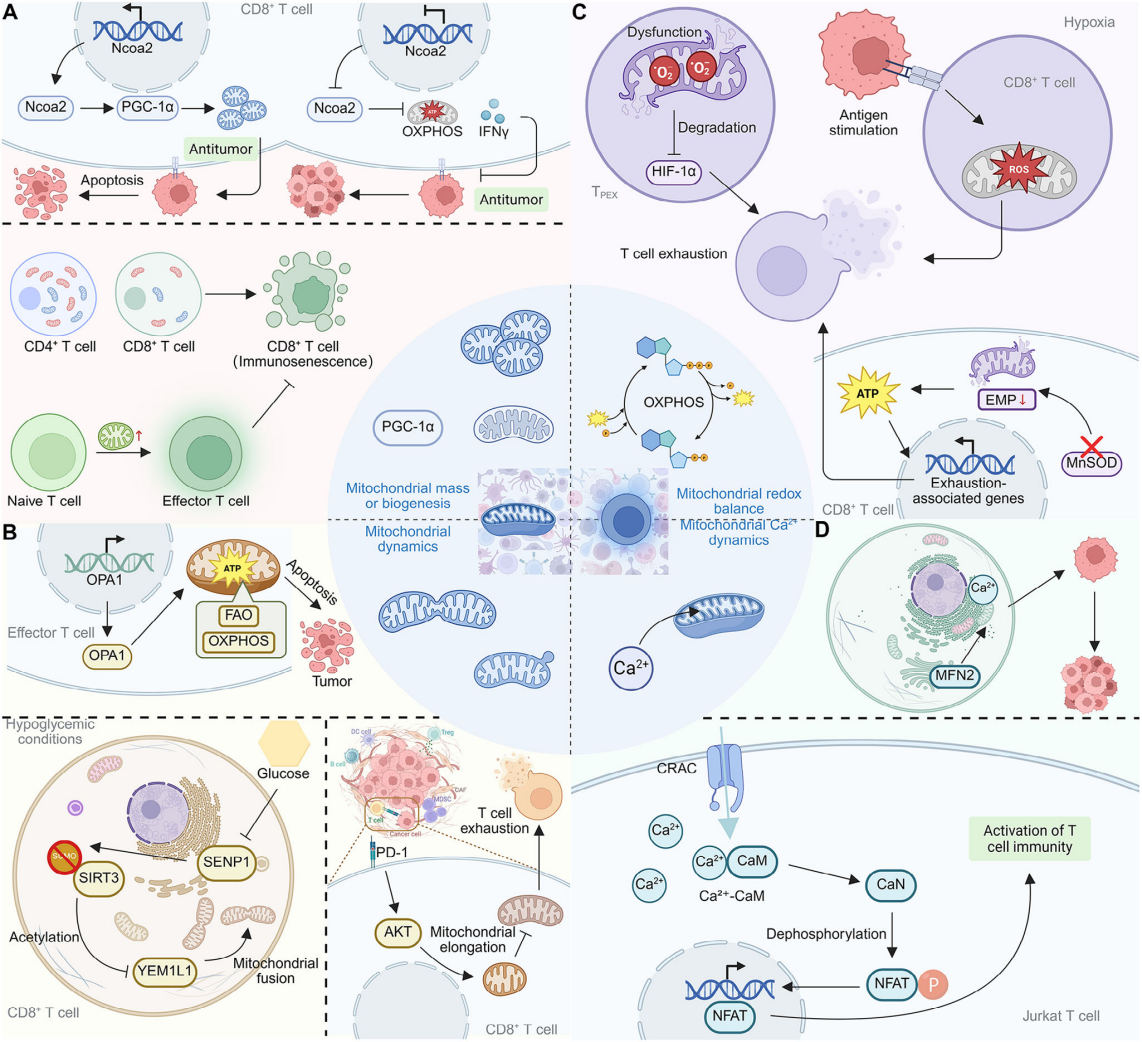
Mitochondrial regulation of CD8⁺ T cell function and exhaustion. A) Ncoa2 regulates PGC‐1α expression, enhancing OXPHOS and IFN‐γ production in CD8⁺ T cells, promoting antitumor immunity while preventing apoptosis. T cell differentiation progresses from naïve T cells to effector T cells, with aged CD8⁺ T cells exhibiting immunosenescence, impairing antitumor responses. B) OPA1 supports mitochondrial dynamics by maintaining FAO and OXPHOS, preventing apoptosis in effector T cells within the TME. Under hypoglycemic conditions, SIRT3‐SENP1‐YME1L1 signaling enhances mitochondrial fusion, sustaining CD8⁺ T cell function. However, PD‐1‐mediated AKT activation leads to mitochondrial elongation, contributing to T‐cell exhaustion. C) Hypoxia and persistent antigen stimulation induce ROS accumulation in CD8⁺ T cells, driving exhaustion through mitochondrial dysfunction. HIF‐1α‐mediated metabolic reprogramming downregulates MnSOD, promoting oxidative stress. Reduced EMP and ATP production further activate exhaustion‐associated genes, impairing T cell function. D) MFN2 regulates mitochondrial‐ER contacts, modulating Ca^2^⁺ homeostasis in T cells. CRAC channels facilitate Ca^2^⁺ influx, activating CaM‐CaN signaling and NFAT dephosphorylation, leading to T‐cell activation.

Mitochondrial mass or biogenesis is a critical determinant of CD8⁺ T cell function, regulating their energy production, proliferation, and effector responses to ensure optimal performance in immune responses. For instance, CD8⁺ T cells exhibit faster immunosenescence than CD4⁺ T cells due to lower mitochondrial content. This mitochondrial deficit in CD8⁺ T cells limits their nutrient uptake, metabolic versatility, and function. Reducing mitochondrial function in CD4⁺ T cells induces a CD8⁺‐like senescent phenotype, highlighting the role of mitochondrial mass in governing T cell senescence.^[^ ^29^ ^]^ Similarly, memory CD8⁺ T cells possess increased mitochondrial mass compared to naïve cells, enhancing both their oxidative and glycolytic capacities upon activation. This mitochondrial advantage supports faster proliferation, increased cytokine production, and sustained ATP levels, essential for rapid response to reinfection.^[^ ^30^ ^]^ In PD‐1 blockade therapy, increased mitochondrial mass and ROS production in tumor‐reactive CD8⁺ T cells within draining lymph nodes enhance T cell expansion and antitumor activity when combined with activation of mTOR, AMPK, or PGC‐1α. This highlights the critical role of mitochondrial mass in boosting the effectiveness of immunotherapy.^[^ ^31^ ^]^ Importantly, overexpression of PGC‐1α, a key regulator of mitochondrial biogenesis, enhances CD8⁺ T cell central memory formation and metabolic fitness, leading to stronger antitumor immunity.^[^ ^32^ ^]^ Likewise, early mitochondrial biogenesis is crucial for the differentiation of naive CD8⁺ T cells into effector cells, driving increased mitochondrial mass, respiration, and mROS production. This process is essential for cytokine production, including IL‐2, TNF, IFN‐γ, perforin, and granzyme B. Reprogramming T cells with enforced PGC1α expression restores their metabolic fitness and enhances antitumor activity, highlighting mitochondrial biogenesis as a key target for reinvigorating dysfunctional T cells in cancer therapy.^[^ ^33^ ^]^ Moreover, Ncoa2 enhances CD8⁺ T‐cell antitumor responses by upregulating PGC‐1α, thus boosting mitochondrial biogenesis and T‐cell activation. Deficiency in Ncoa2 impairs T‐cell oxidative phosphorylation and IFN‐γ production, diminishing tumor rejection capabilities (**Figure** **3**A).^[^ ^34^ ^]^ In chronic lymphocytic leukemia (CLL), CD8^+^ T cells display metabolic reprogramming with diminished GLUT1 reserves and altered mitochondrial dynamics, correlating with impaired activation. Enhanced mitochondrial biogenesis in CD8^+^ CAR T cells, demonstrated by increased mitochondrial mass, correlates positively with the efficacy of CAR T‐cell therapy, suggesting that enhancing mitochondrial biogenesis could potentiate cellular immunotherapies in CLL.^[^ ^35^ ^]^ Crucially, CD8⁺ T cells in juvenile SLE exhibit reduced cytotoxicity, linked to heightened type I interferon signaling, mitochondrial dysfunction, and metabolic imbalance. These cells display mitochondrial enlargement and increased IFN‐α sensitivity, leading to selective loss of effector memory subsets enriched in cytotoxic mediators. The findings highlight mitochondrial impairment as a central driver of cytotoxic T‐cell dysfunction in IFN‐driven autoimmunity.^[^ ^36^ ^]^ Although enhancing mitochondrial mass and biogenesis has shown promise in improving CD8⁺ T cell function, most supporting data rely on enforced gene overexpression or murine models, which may not fully translate to human settings. The balance between promoting bioenergetic fitness and avoiding excessive ROS production or premature differentiation remains a critical concern. Additionally, few studies address whether increased mitochondrial content alone is sufficient to sustain long‐term T cell persistence and function in the TME. Further work is needed to define optimal thresholds and context‐specific effects of mitochondrial augmentation.

### 4.2 Mitochondrial Dynamics in the Regulation of CD8⁺ T Cell Fate and Function

Mitochondrial dynamics describes the continuous remodeling of mitochondrial morphology and function through balanced fusion and fission events. Fusion promotes mitochondrial function and resistance to apoptosis, whereas fission facilitates the removal of damaged mitochondria. Proper regulation of mitochondrial dynamics maintains cellular metabolism, redox equilibrium, and overall homeostasis, and dysregulation contributes to disease pathogenesis and immune dysfunction. Therefore, mitochondrial dynamics, such as fusion and fission, play crucial roles in regulating the metabolic programming of T cells.^[^ ^37^ ^]^ Effector T cells exhibit punctate mitochondria favoring aerobic glycolysis, while memory T cells have fused mitochondrial networks that enhance OXPHOS and FAO via cristae restructuring.^[^ ^18^ ^]^ The fusion protein OPA1 is essential for maintaining memory T cell metabolism and enhancing their antitumor function, highlighting mitochondrial remodeling as a key determinant in T cell fate and function.^[^ ^38^ ^]^ Similarly, CD137 costimulation in CD8⁺ T cells, via agonist mAb or CD137L, enhances mitochondrial mass, potential, and respiratory capacity by promoting OPA1‐dependent mitochondrial fusion. This fusion is crucial for the enhanced antitumor activity of CD8⁺ T cells in murine and human models, underscoring the role of mitochondrial dynamics in improving CD8⁺ T cell function during cancer immunotherapy.^[^ ^13^ ^]^ Additionally, SENP1 promotes CD8⁺ T cell memory development by deSUMOylating SIRT3, enhancing its deacetylase activity, which drives OXPHOS and mitochondrial fusion. This process involves reduced acetylation of YME1L1, leading to mitochondrial fusion and improved T‐cell survival.^[^ ^39^ ^]^ Deletion of the mitochondrial fission protein DRP1 in CD8⁺ T cells promotes mitochondrial elongation, leading to a disproportionate increase in memory T cell populations. Despite compromised primary effector function, DRP1‐deficient memory CD8⁺ T cells mount effective secondary responses. The formation of memory cells in the absence of DRP1 is linked to reduced T‐cell receptor expression rather than altered differentiation or survival. Interestingly, PD‐1 signaling in tumor‐infiltrating CD8⁺ T cells downregulates DRP1 activity, resulting in mitochondrial elongation and reduced cellular motility and proliferation. This regulatory effect on mitochondrial fission highlights a potential therapeutic target within the mitochondrial metabolism of CD8⁺ T cells to enhance anticancer immune responses.^[^ ^40^ ^]^ Notably, persistent metabolic stress in tumors causes depolarized mitochondria to accumulate in tumor‐infiltrating CD8⁺ T cells, driving terminal exhaustion. TCR stimulation, microenvironmental stress, and PD‐1 signaling exacerbate mitochondrial dysfunction, inducing epigenetic changes that reinforce exhaustion. Enhancing mitochondrial fitness with nicotinamide riboside (NR) improves responses to anti‐PD‐1 therapy, emphasizing the role of mitochondrial dynamics in T cell function and exhaustion in cancer (Figure 3B).^[^ ^41^ ^]^ While mitochondrial dynamics are increasingly recognized as a key determinant of T cell metabolic fate, its temporal plasticity and reversibility of fusion‐fission transitions remain poorly defined, especially under chronic stimulation or checkpoint blockade. Mitochondrial plasticity represents the adaptive capability of mitochondria to dynamically alter their morphology, positioning, and function in response to changing cellular demands. This plasticity enables mitochondria to adjust to intracellular and extracellular signals, thus maintaining metabolic efficiency and cellular resilience. Additionally, the extent to which modulating dynamics can be leveraged therapeutically without disrupting broader metabolic or epigenetic programs is unclear. A more nuanced understanding of how mitochondrial morphology integrates with signaling and transcriptional networks will be essential for translating these findings into effective immunomodulatory strategies.

### 4.3 Mitochondrial Redox Balance in the Regulation of CD8⁺ T Cell Activation and Exhaustion

Redox balance, which denotes the equilibrium between oxidation and reduction reactions within biological systems, is crucial for maintaining cellular function and homeostasis. By regulating metabolic processes, signal transduction, and antioxidative responses, redox balance prevents oxidative damage caused by ROS and supports cellular integrity. Mitochondrial redox balance is essential for T cell function, as it governs energy production, ROS regulation, and metabolic adaptability, all of which are critical for supporting their activation, proliferation, and effector responses. T cells from mice lacking complex III ROS production show impaired antigen‐specific expansion despite normal bioenergetic capabilities, indicating a specific need for ROS‐dependent signaling in T cell activation. This highlights the essential role of mitochondrial‐derived ROS in modulating T‐cell responses and functional maturation.^[^ ^12^ ^]^ Notably, mitochondrial dysfunction in T cells triggers redox stress, inhibiting the degradation of HIF‐1α and driving the metabolic transition of precursor‐exhausted T cells (Tpex) to terminally exhausted states.^[^ ^42^ ^]^ Likewise, continuous antigenic stimulation and hypoxia collaboratively drive CD8⁺ T cell exhaustion by impairing mitochondrial function and increasing ROS. This metabolic stress inhibits mitochondrial reprogramming and enhances dysfunction through nuclear factor activation. Mitigating ROS and modifying hypoxia conditions enhance T cell functionality and synergize with immunotherapy, underscoring the critical role of mitochondrial redox balance in shaping T cell fate and effectiveness in disease environments (Figure 3C).^[^ ^43^ ^]^ Additionally, chronic antigenic stimulation impairs CD8⁺ T cell self‐renewal by disrupting ADP‐coupled oxidative phosphorylation, leading to reduced ATP production and proliferation. This mitochondrial dysfunction upregulates exhaustion‐associated genes. Preventing oxidative stress during chronic stimulation sustains T cell proliferation, induces stem‐like progenitor genes, and enhances antitumor immunity.^[^ ^44^ ^]^ Moreover, elevated mitochondrial superoxide in MnSOD‐deficient CD8⁺ T cells disrupts both mitochondrial and glycolytic metabolism, reducing ATP production and impairing T cell activation. This redox imbalance leads to dysregulated cytokine production, increased apoptosis, and altered DNA methylation, affecting key cellular processes necessary for proper T‐cell function (Figure 3C).^[^ ^45^ ^]^ In clear cell renal cell carcinoma (ccRCC), CD8⁺ TILs are metabolically impaired, characterized by inefficient glucose uptake, defective glycolysis, and hyperpolarized, fragmented mitochondria with elevated ROS due to downregulated SOD2. These mitochondrial defects hinder TIL activation, but providing pyruvate or scavenging ROS can partially restore their function.^[^ ^46^ ^]^ In addition, disruption of mitochondrial oxidative phosphorylation accelerates CD8⁺ T cell exhaustion and impairs antitumor immunity. Pharmacological inhibition of the electron transport chain or CRISPR‐mediated deletion of the complex I subunit NDUFA10 compromises mitochondrial fitness, enhancing exhaustion and tumor progression. These findings underscore the essential role of oxidative metabolism in sustaining CD8⁺ T cell function within the TME.^[^ ^47^ ^]^ Interestingly, elevated mitochondrial ROS in CD8^+^ T cells is observed in young adults with major depressive disorder (MDD), suggesting early immune dysregulation. This increase in mitochondrial ROS occurs without corresponding elevations in circulating proinflammatory cytokines, highlighting a potential early marker of immune system impairment in otherwise healthy individuals with MDD.^[^ ^48^ ^]^ Notably, the long‐chain fatty acid eicosenoate impairs CD8^+^ T cell function in early HIV infection by increasing mitochondrial ROS and decreasing mitochondrial function, mediated through p53 induction. This metabolic dysregulation leads to reduced T‐cell proliferation, cytokine secretion, and increased TIM‐3 expression. Targeting mitochondrial ROS with mito‐TEMPO restores T cell function, highlighting a potential therapeutic strategy for immune restoration in HIV infection.^[^ ^49^ ^]^ While the role of redox homeostasis in CD8⁺ T cell function is increasingly appreciated, current studies often fail to delineate the boundary between physiological ROS signaling and pathological oxidative stress. In particular, many rely on extreme genetic models (e.g., complete MnSOD deficiency), which may not accurately reflect subtle, progressive redox imbalances observed in human disease. Additionally, the interface between mitochondrial ROS and epigenetic regulation remains insufficiently characterized, despite its likely relevance in shaping exhaustion phenotypes. A more nuanced understanding of redox thresholds and context‐specific sensitivity is needed to guide the design of interventions that restore mitochondrial function without disrupting essential redox signaling.

### 4.4 Mitochondrial Calcium Dynamics in the Regulation of CD8⁺ T Cell Activation and Signaling

Mitochondrial calcium signaling encompasses the precise regulation of calcium ion concentrations within mitochondria, critically influencing mitochondrial metabolism, energy production, and cellular responses to stress. Calcium influx via channels such as the mitochondrial calcium uniporter modulates oxidative phosphorylation, energy metabolism, and cellular survival pathways, thereby safeguarding cellular homeostasis. Mitochondrial regulation of calcium dynamics is integral to T cell activation and function, orchestrating intracellular signaling pathways through precise control of calcium influx and organelle cross‐talk. This fine‐tuned interaction not only supports immune responses but also highlights potential therapeutic targets in conditions where calcium homeostasis is disrupted. In Jurkat T cells, mitochondria maintain CRAC channel activity by preventing local Ca^2+^ accumulation, which can inactivate these channels. Disruption of mitochondrial Ca^2+^ uptake impairs CRAC functionality and T‐cell activation by affecting the nuclear translocation of NFAT.^[^ ^50^ ^]^ Similarly, mitochondrial positioning enhances local calcium entry through ORAI channels in T cells by preventing channel inactivation. Upon forming an immunological synapse (IS), mitochondria migrate to the IS, boosting calcium influx essential for T cell activation. This localized calcium modulation, independent of ORAI channel concentration at the IS, highlights a sophisticated mechanism where mitochondrial placement dictates T‐cell signaling and functionality.^[^ ^51^ ^]^ In addition, mitofusin‐2 (MFN2) boosts mitochondrial metabolism in CD8⁺ T cells by enhancing mitochondria‐endoplasmic reticulum contacts, thereby facilitating mitochondrial calcium influx via SERCA2 interactions. This organelle cross‐talk prevents excessive calcium accumulation and apoptosis, improving the metabolic fitness and efficacy of tumor‐infiltrating CD8⁺ T cells in cancer immunotherapy.^[^ ^52^ ^]^ Notably, bile acids in cholestasis disrupt T cell function by impairing intracellular calcium homeostasis, crucial for NFAT signaling and activation. This is mechanistically mediated through the inhibition of mitochondrial calcium uptake and the resulting elevated cytoplasmic Ca^2+^ levels, decoupling STIM1 and ORAI1 and impairing store‐operated Ca^2+^ entry. This dysfunction leads to poor viral clearance in HBV‐infected mice, highlighting a potential therapeutic target in bile acid regulation to enhance T cell‐mediated anti‐viral responses (Figure 3D).^[^ ^53^ ^]^

While current findings underscore the critical role of mitochondrial Ca^2^⁺ handling in sustaining NFAT signaling and T‐cell activation, they largely treat calcium regulation as an isolated signaling axis. However, mitochondrial calcium influx also intersects with metabolic adaptation, apoptotic sensitivity, and mitochondrial ROS production—dimensions that remain insufficiently integrated in the context of T‐cell fate programming. It remains unclear whether targeting mitochondrial Ca^2^⁺ flux can selectively enhance T cell activation without inadvertently promoting metabolic stress or activation‐induced cell death. Furthermore, how mitochondrial‐ER calcium transfer is fine‐tuned during chronic antigen exposure or in suppressive microenvironments is poorly defined. Elucidating these dynamic interfaces may offer a route to reprogram exhausted or dysfunctional CD8⁺ T cells without compromising viability or systemic calcium homeostasis.

### 4.5 Mitochondrial Metabolism in CD8⁺ T Cell Immune Response and Dysfunction

**Figure 4:**
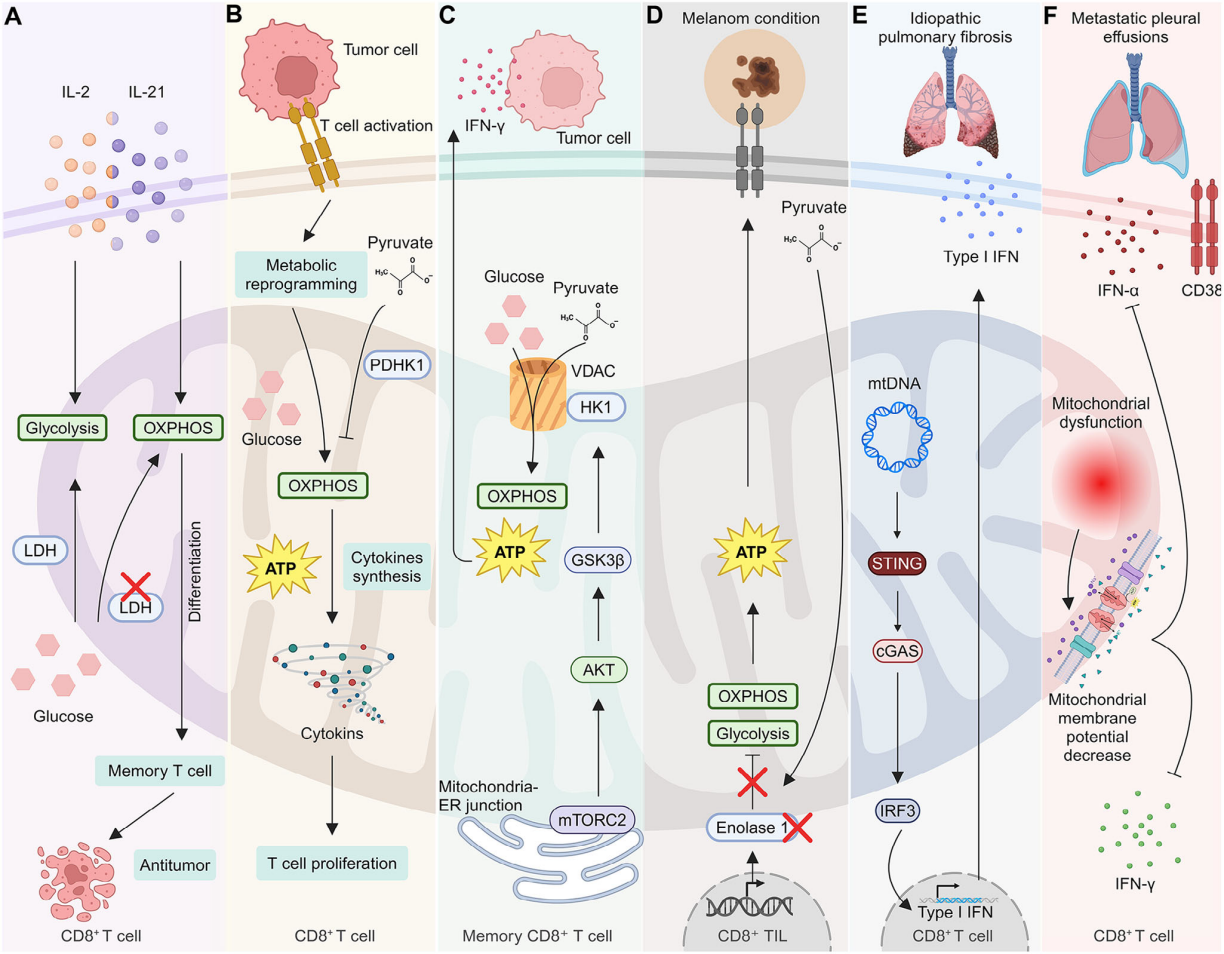
Mitochondrial metabolism in CD8⁺ T cell immune response and dysfunction. A) IL‐2 and IL‐21 orchestrate CD8⁺ T cell differentiation by modulating metabolic pathways. IL‐2 promotes glycolysis, while IL‐21 enhances OXPHOS, favoring memory CD8⁺ T cell formation and antitumor immunity. LDH downregulation shifts metabolism toward OXPHOS, supporting long‐term T‐cell persistence. B) TCR activation triggers metabolic reprogramming in CD8⁺ T cells, enhancing OXPHOS and ATP production through PDHK1‐mediated pyruvate metabolism, thereby promoting cytokine synthesis and proliferation. C) Memory CD8⁺ T cells rely on mitochondrial metabolism, with HK1‐VDAC interactions regulating glucose flux toward OXPHOS at the mitochondria‐ER junction. mTORC2‐AKT‐GSK3β signaling further enhances ATP generation and sustains T cell longevity. D) In melanoma, CD8⁺ TILs depend on OXPHOS for energy production. Enolase inhibition disrupts glycolysis, impairing metabolic flexibility and T‐cell function. E) In idiopathic pulmonary fibrosis, mtDNA leakage activates the cGAS‐STING‐IRF3 pathway, driving type I IFN responses in CD8⁺ T cells. (F) In metastatic pleural effusions, chronic antigen stimulation and inflammatory signals induce CD38 expression, leading to mitochondrial dysfunction, loss of mitochondrial membrane potential, and reduced IFN‐γ production, contributing to CD8⁺ T cell dysfunction.

T cell activation and differentiation are intricately governed by metabolic rewiring, which enables the immune cells to meet the energetic demands of effector functions and long‐term memory formation. For instance, IL‐2 and IL‐21 shape CD8⁺ T cell differentiation through distinct metabolic pathways, with IL‐2 driving glycolysis and terminal differentiation, while IL‐21 promotes oxidative phosphorylation and stem cell memory T cells. Inhibiting lactate dehydrogenase (LDH) shifts IL‐2‐induced metabolism toward oxidative phosphorylation, enhancing memory cell formation and antitumor responses (**Figure** **4**A).^[^ ^54^ ^]^ Also, elevated methylmalonic acid (MMA), associated with aging and vitamin B12 deficiency, induces exhaustion in CD8⁺ T cells by disrupting NADH‐regenerating TCA cycle reactions and impairing mitochondrial function. MMA triggers TOX‐driven transcriptional reprogramming, upregulates exhaustion markers, and suppresses effector activity, thereby weakening anti‐tumor immunity and linking metabolic dysregulation to immune dysfunction in cancer.^[^ ^55^ ^]^ Additionally, in human CD4⁺ and CD8⁺ T cells, glycolysis and mitochondrial activity dynamically change upon activation, impacting proliferation and cytokine production. Mitochondria coordinate glycolytic and oxidative metabolism and play a pivotal role in regulating T cell fate and function, particularly in the context of immune responses against tumors.^[^ ^56^ ^]^ For instance, T‐cell activation triggers aerobic glycolysis by rapidly inducing pyruvate dehydrogenase kinase 1 (PDHK1), which blocks mitochondrial pyruvate import, diverting it to lactate production. This metabolic switch is crucial for cytokine synthesis but not for cytotoxicity. Inhibition of PDHK1 disrupts lactate dehydrogenase‐mediated regulation of cytokine mRNA translation (Figure 4B).^[^ ^57^ ^]^ In addition, mitochondrial pyruvate carrier (MPC) deletion in CD8⁺ T cells promotes differentiation toward a memory phenotype through enhanced metabolic flexibility, favoring acetyl‐coenzyme‐A production and histone acetylation. In the TME, however, MPC supports lactate oxidation critical for antitumor activity. Inhibiting MPC during CAR T cell manufacturing enhances their long‐term antitumor function, indicating that mitochondrial pyruvate uptake is pivotal for T cell differentiation and effective cancer immunotherapy.^[^ ^58^ ^]^ Mitochondria‐ER junctions are specialized interorganelle junctions between mitochondria and the endoplasmic reticulum. These sites facilitate calcium signaling, lipid transfer, and metabolic coordination, significantly influencing cellular metabolism, signaling pathways, and overall homeostasis.^[^ ^59^ ^]^ For instance, rapid activation of mTORC2‐AKT signaling at mitochondria‐ER junctions in memory CD8⁺ T cells inhibit GSK3β, facilitating the recruitment of hexokinase I to VDAC on mitochondria. This promotes glucose metabolism and pyruvate oxidation, crucial for swift IFN‐γ production. This subcellular organization enables the metabolic reprogramming required for the rapid effector function of memory CD8⁺ T cells (Figure 4C).^[^ ^60^ ^]^ Furthermore, P4HA1 accumulation in CD8⁺ T cells impairs mitochondrial metabolism by disrupting the TCA cycle, driving exhaustion, and suppressing progenitor expansion in tumors and tumor‐draining lymph nodes. Inhibiting P4HA1 restores TCF1⁺ progenitor pools and mitochondrial fitness, enhancing both adoptive and endogenous systemic CD8⁺ T cell immunity, and offering a promising target to overcome immune escape in solid tumors.^[^ ^61^ ^]^ Notably, CD8⁺ TILs in melanomas exhibit metabolic dysfunction, marked by impaired glycolysis and oxidative phosphorylation due to downregulated enolase 1 activity. Although enolase 1 mRNA and protein levels are high, its enzymatic activity is repressed post‐translationally. Providing pyruvate bypasses this glycolytic block, enhancing both glycolysis and mitochondrial function, thereby restoring CD8⁺ TIL effector activity (Figure 4D).^[^ ^62^ ^]^ Deletion of the transcriptional repressor Zbtb20 enhances mitochondrial and glycolytic metabolism in CD8⁺ T cells, increasing their metabolic flexibility and spare respiratory capacity. This reprogramming promotes a memory phenotype, leading to stronger secondary responses and improved tumor protection.^[^ ^63^ ^]^ Interestingly, the TCA cycle drives CD8⁺ T cell motility in 3D tumor environments, with mitochondrial oxidation of glucose and glutamine being crucial for migration, while glycolysis plays a lesser role. Enhancing mitochondrial activity improves T cell infiltration and CAR T cell recruitment into tumor islets, resulting in better tumor control in human xenograft models.^[^ ^64^ ^]^

Mitochondrial‐mediated lipid metabolism also plays a pivotal role in regulating CD8⁺ T cell function. Deletion or inhibition of protein tyrosine phosphatase, mitochondrial 1 (PTPMT1) disrupts mitochondrial metabolic flexibility in CD8⁺ T cells, leading to reduced effector development, accelerated exhaustion, and increased tumor growth. This is due to a shift from pyruvate to fatty acid utilization, causing oxidative stress and cellular damage.^[^ ^65^ ^]^ In addition, in obesity‐associated breast cancer, activated STAT3 drives increased FAO in CD8⁺ T cells, suppressing glycolysis and impairing T cell effector functions. This metabolic shift, further exacerbated by PD‐1 signaling and leptin from adipose tissue, promotes tumor progression.^[^ ^66^ ^]^ In pancreatic ductal adenocarcinoma, CD8⁺ T cells accumulate LCFAs in the lipid‐rich TME, impairing mitochondrial function and downregulating fatty acid metabolism. Specifically, reduced expression of VLCAD exacerbates LCFA accumulation, leading to lipotoxicity. Enforcing ACADVL expression in CD8⁺ T cells enhances their survival and persistence, offering a strategy to overcome metabolic challenges and improve immunotherapy outcomes in pancreatic cancer.^[^ ^67^ ^]^

Mitochondrial ATP synthesis is essential for regulating T cell activation, functionality, and immune responses. Upon activation, T cells rapidly enhance mitochondrial ATP production, which is crucial for autocrine purinergic signaling and subsequent cellular functions such as intracellular Ca^2+^ signaling, CD69 expression, and IL‐2 transcription. This spike in ATP also leads to its release into the extracellular space, supporting further T cell activation. Inhibiting mitochondrial function disrupts these processes, underlining the essential role of mitochondrial metabolism in regulating T‐cell activity and host defense mechanisms.^[^ ^68^ ^]^ Similarly, mitochondrial function, including membrane potential and ATP production, is crucial for CD8⁺ T cell activation, migration, and effector function in tumors. Enhanced mitochondrial metabolism correlates with increased granzyme B and IFN‐γ production. Using the “OptoMito‐On” optogenetic tool to boost mitochondrial ATP production improved T cell migration and antitumor activity, highlighting the pivotal role of mitochondria in CD8⁺ T cell‐mediated tumor immunity.^[^ ^69^ ^]^ Despite growing evidence supporting the central role of mitochondrial metabolism in CD8⁺ T cell function, current studies often examine individual metabolic pathways in isolation, without fully elucidating the spatiotemporal integration of glycolysis, OXPHOS, and lipid metabolism during distinct phases of T cell activation and exhaustion. Moreover, although enhancing specific metabolic axes—such as FAO or pyruvate flux—can improve T cell fitness in certain contexts, these interventions may prove detrimental in nutrient‐deprived or lipid‐rich environments like the TME. A more comprehensive, systems‐level understanding of how mitochondrial metabolism is dynamically regulated across diverse immunological landscapes will be critical for developing durable and context‐sensitive therapeutic strategies.

### 4.6 Mitochondrial Autophagy in the Regulation of CD8⁺ T Cell Functional Fitness

Mitophagy is a selective autophagic process that specifically targets dysfunctional or damaged mitochondria for degradation. This mechanism involves the recognition of mitochondrial damage signals, encapsulation within autophagosomes, and subsequent fusion with lysosomes for degradation. Mitophagy maintains mitochondrial integrity, prevents excessive ROS generation, and averts apoptosis, thereby sustaining cellular homeostasis.^[^ ^70^ ^]^ In highly metabolic cells such as CD8⁺ T lymphocytes, mitochondrial health is essential for sustaining energy production, redox balance, and critical cell fate decisions, making mitophagy indispensable for bioenergetic fitness and function. Under physiological conditions, mitophagy significantly shapes CD8⁺ T cell functionality. Naïve CD8⁺ T cells display high basal autophagic flux, with mitophagy maintaining mitochondrial quality and supporting molecules critical for survival and migration.^[^ ^71^ ^]^ During T‐cell differentiation, autophagy is dynamically modulated by antigenic and cytokine signals, orchestrating metabolic adaptation and proteomic remodeling. Specifically, Pink1‐dependent mitophagy in T(SCM) facilitates mitochondrial remodeling and biogenesis, enhancing self‐renewal and persistence via Wnt pathway activation.^[^ ^72^ ^]^ Additionally, NIX‐dependent mitophagy ensures effective memory formation in effector CD8⁺ T cells by preserving metabolic flexibility. Loss of NIX disrupts mitochondrial metabolism, triggering HIF‐1α accumulation and shifting metabolism from long‐chain fatty acid oxidation to less efficient short‐ or branched‐chain fatty acid oxidation, thus impairing ATP synthesis and memory formation phenotype reversible upon metabolic restoration.^[^ ^73^ ^]^ Aberrant mitophagy has been increasingly associated with pathological immune states. For instance, impaired Parkin‐dependent mitophagy accelerates skin‐graft rejection by promoting effector differentiation and inflammatory cytokine production in CD8⁺ T cells, effects mitigated by pharmacological activation of mitophagy.^[^ ^74^ ^]^ In colorectal cancer, hypercholesterolemia‐induced ER stress enhances ER‐mitochondria interactions, driving excessive mitophagy and mitochondrial energy disturbances, culminating in CD8⁺ T cell exhaustion and immune evasion.^[^ ^75^ ^]^ Collectively, these findings emphasize the critical importance of precisely regulated mitophagy for sustaining CD8⁺ T cell function across physiological and pathological contexts. Moreover, metabolic reprogramming of CAR T cells using semaglutide and urolithin A enhances autophagy and mitophagy via mTOR inhibition and Atg4b activation, sustaining mitochondrial fitness and promoting CD8⁺ T cell memory. Engineered GLP‐1‐secreting CAR T cells maintain stemness, persistence, and anti‐tumor activity while reducing cytokine release syndrome, underscoring the therapeutic potential of targeting mitophagy to improve CAR T cell durability and function.^[^ ^76^ ^]^ Notably, ammonia accumulation in proliferating effector T cells disrupts lysosomal homeostasis and triggers mitochondrial damage, leading to a distinct form of cell death marked by lysosomal alkalization, mitochondrial swelling, and impaired autophagic flux. Inhibiting glutaminolysis or lysosomal alkalization preserves T cell viability and enhances antitumor efficacy, highlighting a metabolic vulnerability that can be targeted to improve T cell‐based immunotherapy.^[^ ^77^ ^]^

### 4.7 Mitochondrial Transfer as a Bidirectional Switch for CD8⁺ T Cell Function

Mitochondrial transfer emerges as a critical regulator of CD8^+^ T cell functionality across diverse pathological conditions. In autoimmune diseases and transplant rejection, MSCs suppress pathogenic CD8^+^ T cell activation by transferring mitochondria, resulting in diminished T cell proliferation, IFN‐γ production, and reduced diabetogenic potential. Mechanistically, this immunosuppressive effect involves the downregulation of key transcription factors T‐bet and Eomes, in synergy with MSC‐secreted prostaglandin E₂.^[^ ^78^ ^]^ Importantly, inhibition of mitochondrial transfer attenuates these suppressive outcomes, underscoring mitochondrial transfer as a clinically relevant therapeutic mechanism for immune‐mediated diseases. Conversely, mitochondrial transfer can enhance CD8^+^ T cell function in cancer immunotherapy contexts. Bone marrow stromal cells transfer mitochondria to CD8^+^ T cells via nanotubular connections, a process dependent on Talin 2 expression in both donor and recipient cells. This transfer augments mitochondrial respiration and spare respiratory capacity, thereby promoting robust T‐cell expansion, improved tumor infiltration, and resistance to exhaustion. Consequently, mitochondria‐enriched CD8^+^ T cells exhibit superior antitumor efficacy and prolong survival in tumor‐bearing models.^[^ ^79^ ^]^ Moreover, mitochondrial transfer inhibition has been shown to prevent cancer cells from hijacking mitochondria from immune cells, thus potentiating antitumor immunity. Specifically, in a syngeneic 4T1 breast tumor model, the mitochondrial transfer inhibitor L‐778123 enhances CD8^+^ T cell tumor infiltration and significantly improves the efficacy of immune checkpoint inhibitors.^[^ ^80^ ^]^ Collectively, these findings establish mitochondrial transfer as a promising and actionable therapeutic target for next‐generation immunotherapeutic strategies.

### 4.8 Emerging Mitochondrial‐Mediated Mechanisms Regulating CD8⁺ T Cell Immunity

Accumulating evidence has shown that mitochondria play a pivotal role in regulating CD8⁺ T cell functions through a variety of emerging mechanisms.^[^ ^81^, ^82^ ^]^ For instance, mitochondrial DNA stress activates the immunoproteasome via the cGAS/STING/type I IFN pathway, enhancing MHC class I antigen presentation and autonomously activating CD8⁺ T cells.^[^ ^83^ ^]^ This mechanism, also triggered by genomic DNA stress, is observed in both epithelial and mesenchymal cells across species. In idiopathic pulmonary fibrosis, this pathway contributes to CD8⁺ T cell activation in lung tissues, offering new therapeutic targets for modulating immune responses in pulmonary disorders (Figure 4E).^[^ ^84^ ^]^ Interestingly, mitochondrial translation, the synthesis of mitochondrial proteins mediated by mitochondrial ribosomes within mitochondria, is essential for producing key components of the electron transport chain and oxidative phosphorylation, thereby sustaining cellular energy production, continuous protein synthesis, and replenishment of cytotoxic effectors necessary for serial CTL killing. Acute mitochondrial depletion or inhibition of mitochondrial translation impairs CTL function, highlighting mitochondria's role as a key regulator of CTL‐mediated cytotoxicity.^[^ ^85^ ^]^ In addition, CD38 expression on CD8⁺ T cells in metastatic pleural effusions correlates with increased PD‐1 levels and an exhausted phenotype, characterized by diminished IFNγ and TNFα production and reduced mitochondrial membrane potential. This suggests that mitochondrial dysfunction within CD38⁺CD8⁺ T cells contributes to their impaired immune response, highlighting a potential target for therapeutic intervention in tumor environments (Figure 4F and **Table** **1**).^[^ ^41^ ^]^

**Table 1:** Targeting mitochondria for improving CD8^+^ T cell‐based immunotherapy.

Mitochondrial Biology	Mechanism	Biological Impact	Refs.
Mitochondrial mass	Using rotenone to reduce the mitochondrial mass of CD4^+^ T cell	Inhibiting the proliferation and migration of T cells	[29]
	CD8^+^ TM cells have more mitochondrial mass	Enhancing ATP production and improving response to reinfection	[30]
	Activating the mTOR, AMPK, and PGC‐1α signaling pathways increasing mitochondrial mass	Increasing expression of downstream transcription factors	[31]
Mitochondrial biogenesis	Naive CD8⁺ T cell activation enhances mitochondrial biogenesis	Promoting the differentiation of CD8⁺ T cells into effector cells	[33]
	Ncoa2 upregulates PGC‐1α expression to promote mitochondrial biogenesis	Enhancing T‐cell activation, increasing IFN‐γ production	[34]
	Reducing GLUT1 reserves lead to impaired mitochondrial biogenesis	Impairing T‐cell activation, reducing glucose uptake	[35]
Mitochondrial dynamics	OPA1 deficiency results in mitochondrial fragmentation	Defective T cell maturation, inability to generate long‐term memory T cells	[38]
	Promoting mitochondrial fusion by activating the SENP1‐Sirt3 signaling pathway	Enhancing T cell survival, promoting the development of T‐cell memory	[39]
	Reducing Drp1 phosphorylation to inhibit mitochondrial fission	Inhibiting T cell migration, proliferation, and glycolysis	[40]
	Exhibiting reduced mitochondrial membrane potential in CD38^+^CD8^+^ T cells	Decreasing IFN‐γ and TNFα production	[41]
Mitochondrial redox balance	Mitochondrial dysfunction in T cells triggers redox stress	Leading to T‐cell exhaustion	[42]
	Antigen stimulation and hypoxia increase the production of ROS	Reducing cytokine secretion, decreasing cytotoxicity	[43]
	Disrupting ADP‐coupled oxidative phosphorylation to reduce ATP production	Suppressing T cell proliferation	[44]
	Elevating levels of superoxide disrupt glycolysis.	Dysregulating cytokine production, increasing apoptosis	[45]
	Providing pyruvate to bypass glycolysis defects	Partially restoring TIL activation	[46]
	Elevating T cell mitoROS levels	Increasing risk of cardiovascular disease	[48]
	Long‐chain fatty acid arachidonate increases mitoROS	Increasing cytokine secretion and TIM‐3 expression	[49]
Mitochondrial Ca^2+^ dynamics	Antigen stimulation blocks Ca^2+^ uptake	Inhibiting the nuclear translocation of NFAT transcription factors	[50]
	Absorbing Ca^2+^ to maintain the sustained activation of ORAI channels	Enhancing T‐cell activation, sustaining NFAT activity	[51]
	Enhancing mitochondrial‐ER contact to promote Ca^2+^ influx	Increasing CD8^+^ T cell metabolic fitness and function in tumors	[52]
	Impairing intracellular calcium homeostasis bile acids	Inhibiting T cell activation and metabolism	[53]
Mitochondrial metabolism	IL‐21 promotes oxidative phosphorylation	Enhancing memory cell formation	[54]
	Disrupting NADH‐regenerating TCA cycle reactions	Increasing CD8⁺ T cell exhaustion	[55]
	Activating PDHK1 to inhibit pyruvate entry to promote lactate production	Having an acute impact on cytokine synthesis	[57]
	MPC deletion promotes differentiation toward a memory phenotype	Enhancing long‐term antitumor function	[58]
	Promoting glucose metabolism and pyruvate oxidation	Facilitating the rapid production of IFN‐γ	[60]
	Inhibiting P4HA1 restores mitochondrial metabolic adaptation	Enhancing CD8⁺ T cell immunity	[61]
	Down‐regulation of enolase 1 activity represses glycolytic activity	Restraining effector functions of tumor‐infiltrating CD8^+^ T cells	[62]
	Zbtb20 deficiency enhances glycolytic metabolism.	Enhancing spare respiratory capacity in memory CD8^+^ T cells	[63]
	Succinate accumulation inhibits PDH activity.	Increasing risk of apoptosis	[64]
	PTPMT1 deletion impairing mitochondrial metabolic flexibility	Reducing effector development	[65]
	STAT3 drives an increase in FAO	Promoting tumor progression	[66]
	LCFA accumulation downregulates FAO	Driving dysfunction in intrapancreatic CD8^+^ T cells	[67]
	Increasing intracellular ATP levels and release	Regulating T cell function and host defense	[68]
Mitophagy	Preventing HIF1α accumulation	Promoting effector memory formation	[73]
	Regulating ERS‐ERMC‐mitophagy axis	Leading to CRC immune escape	[75]
Mitochondrial transfer	Transfer mitochondria via nanotubular connections	promoting robust T‐cell expansion	[79]
DNA sensing	Activation of DNA stress via cGAS/STING/type I IFN pathway	Enhancing antigen presentation	[83]

Mitochondrial lactylation, a recently characterized post‐translational modification, involves the covalent addition of lactyl groups—derived from intracellular lactate—to lysine residues on mitochondrial proteins, linking metabolic stress to mitochondrial reprogramming and cellular fate.^[^ ^86^ ^]^ This modification has emerged as a hypoxia‐responsive mechanism that suppresses OXPHOS. For instance, under hypoxic conditions, AARS2‐mediated lactylation of PDHA1 and CPT2 limits acetyl‐CoA entry from glycolysis and FAO, while the de‐lactylate SIRT3 reverses this inhibition and dynamically adjusts mitochondrial output during stress.^[^ ^87^ ^]^ Similarly, lactylation of mitochondrial Tufm impairs mitophagy and exacerbates neuronal damage following traumatic brain injury, whereas its inhibition improves neuroprotection.^[^ ^88^ ^]^ In the liver, PGC‐1α protects against acetaminophen‐induced toxicity by enhancing LDHB expression and reducing mitochondrial lactylation, thus preserving mitochondrial quality.^[^ ^89^ ^]^ Furthermore, Dexmedetomidine mitigates myocardial ischemia‐reperfusion injury by reducing MDH2 lactylation, linking this modification to ferroptosis and mitochondrial dysfunction via regulation of PDK4 and glycolytic flux.^[^ ^90^ ^]^ While these studies underscore mitochondrial lactylation as a critical metabolic checkpoint in various disease models, its role in CD8⁺ T cells remains largely undefined. Given that mitochondrial integrity orchestrates effector differentiation, memory formation, and resistance to exhaustion, it is plausible that lactylation of mitochondrial enzymes functions as a metabolic brake under hypoxic or lactate‐enriched tumor microenvironments. Such modification may restrict OXPHOS and ATP production, contributing to T cell dysfunction and impaired antitumor activity. Elucidating this regulatory layer may reveal novel targets to reinvigorate CD8⁺ T cells and enhance immunotherapy efficacy in metabolically hostile tumors.

## 5 Therapeutic Strategies Targeting Mitochondria to Enhance CD8⁺ T Cell‐Based Immunotherapy

Mitochondrial modulation in CD8^+^ T cells has emerged as a promising strategy to enhance the efficacy of immunotherapy.^[^ ^91^ ^]^ Small molecules, including both organic and inorganic compounds, have shown significant potential in modulating mitochondrial function to improve T cell activation, persistence, and cytotoxicity.^[^ ^92^ ^]^ For instance, linoleic acid bolsters CD8⁺ T cell mitochondrial metabolism and effector functions by enhancing ER‐mitochondria contacts, facilitating calcium signaling, and mitochondrial energetics. This metabolic enhancement prevents T cell exhaustion and promotes a memory‐like phenotype, significantly improving antitumor responses in adoptive T cell therapy.^[^ ^93^ ^]^ In addition, Bezafibrate enhances PD‐1 blockade efficacy in lung cancer by promoting FAO in tumor‐infiltrating CD8⁺ T cells. This synergy increases CTL infiltration, survival, and activation within the TME while upregulating FAO‐related genes and CXCL9/CXCL10 expression.^[^ ^94^ ^]^ Similarly, Bezafibrate boosts mitochondrial metabolism in CD8⁺ T cells by upregulating oxidative phosphorylation, glycolysis, and FAO, supporting CTL proliferation, energy needs, and survival.^[^ ^95^ ^]^ Notably, nicotinamide (NAM) modulates mitochondrial metabolism in activated CD8⁺ T cells by reducing mitochondrial content and ROS levels, leading to diminished apoptotic death and enhanced clonal expansion. Despite lower cytokine expression, NAM extends the proliferative capacity of T cells, potentially offering a strategy to increase T cell expansion during activation without depleting their replicative lifespan.^[^ ^96^ ^]^ Remarkably, NR preserves mitochondrial function and attenuates exhaustion in CD8⁺ T cells under chronic stimulation or tumor‐derived stress. NR enhances Sirt1 expression, reducing apoptosis and maintaining metabolic fitness, while pharmacological inhibition of Sirt1 abrogates these effects. These findings highlight the Sirt1‐dependent mitochondrial support of NR as a promising approach to improve T cell persistence and immunotherapy efficacy.^[^ ^97^ ^]^ Likewise, elevated DNA damage and defective repair in HBV‐specific CD8 T cells, driven by NAD depletion and excessive CD38 activity, contribute to T cell exhaustion in chronic HBV infection. NAM mononucleotide supplementation restores mitochondrial function and T‐cell antiviral activity by correcting these metabolic imbalances.^[^ ^98^ ^]^ Spermidine (SPD) supplementation enhances mitochondrial function and CD8⁺ T cell activation in aged mice, overcoming immunotherapy impairments. SPD acutely boosts FAO in CD8⁺ T cells by binding and allosterically activating the mitochondrial trifunctional protein (MTP). This interaction is crucial for SPD's ability to enhance PD‐1 blockade efficacy, demonstrating the dependency of SPD‐mediated T cell activation on MTP function.^[^ ^99^ ^]^ Moreover, Lithium carbonate mitigates lactic acid (LA)‐mediated immunosuppression in CD8⁺ T cells by blocking lysosomal acidification, thus rescuing mitochondrial localization of monocarboxylate transporter 1. This enables mitochondrial oxidation of LA, enhancing CD8⁺ T cell function and potentially supporting cancer immunotherapy by targeting LA metabolism.^[^ ^100^ ^]^ Importantly, many natural compounds and traditional Chinese medicines have also shown significant potential in regulating CD8⁺ T cell mitochondrial modulation, further enhancing their antitumor functions and improving the outcomes of immunotherapy. For instance, in chronic HBV infection, HBV‐specific CD8^+^ T cells exhibit impaired autophagic flux and mitochondrial dysfunction, contributing to T cell exhaustion. Treatment with polyphenols like resveratrol and oleuropein significantly improved mitochondrial function, proteostasis, and antiviral activity in these cells.^[^ ^101^ ^]^ Additionally, these compounds enhanced the efficacy of mitochondria‐targeted antioxidants and PD‐1/PD‐L1 blockade.^[^ ^21^ ^]^ Similarly, oxymatrine (Om) and astragaloside IV (As) enhance the anti‐tumor immune response in TNBC by modulating the TME. Om suppresses cancer‐associated fibroblast activation, promoting T cell infiltration, while as increases mitochondrial quantity and cristae in T cells. The combined treatment optimizes the immune system's ability to attack TNBC, improving T cell infiltration and function, and increasing tumor cell apoptosis.^[^ ^101^ ^]^ Additionally, berberine prolongs allograft survival by inducing apoptosis in alloreactive CD8^+^ T cells through the mitochondrial apoptosis pathway. In a cardiac transplantation model, berberine reduced T cell infiltration and inhibited T cell activation, proliferation, and function, ultimately protecting myocardial cells.^[^ ^102^ ^]^ Interestingly, the Yi‐Qi‐Jian‐Pi formula enhances immune function in a rat model of acute‐on‐chronic liver failure by improving mitochondrial biosynthesis and autophagy in CD8^+^ T lymphocytes. Key mechanisms include the upregulation of PGC‐1α, NRF‐1, and TFAM, which promote mitochondrial biogenesis and functional homeostasis, thereby alleviating immune dysfunction.^[^ ^103^ ^]^

**Figure 5:**
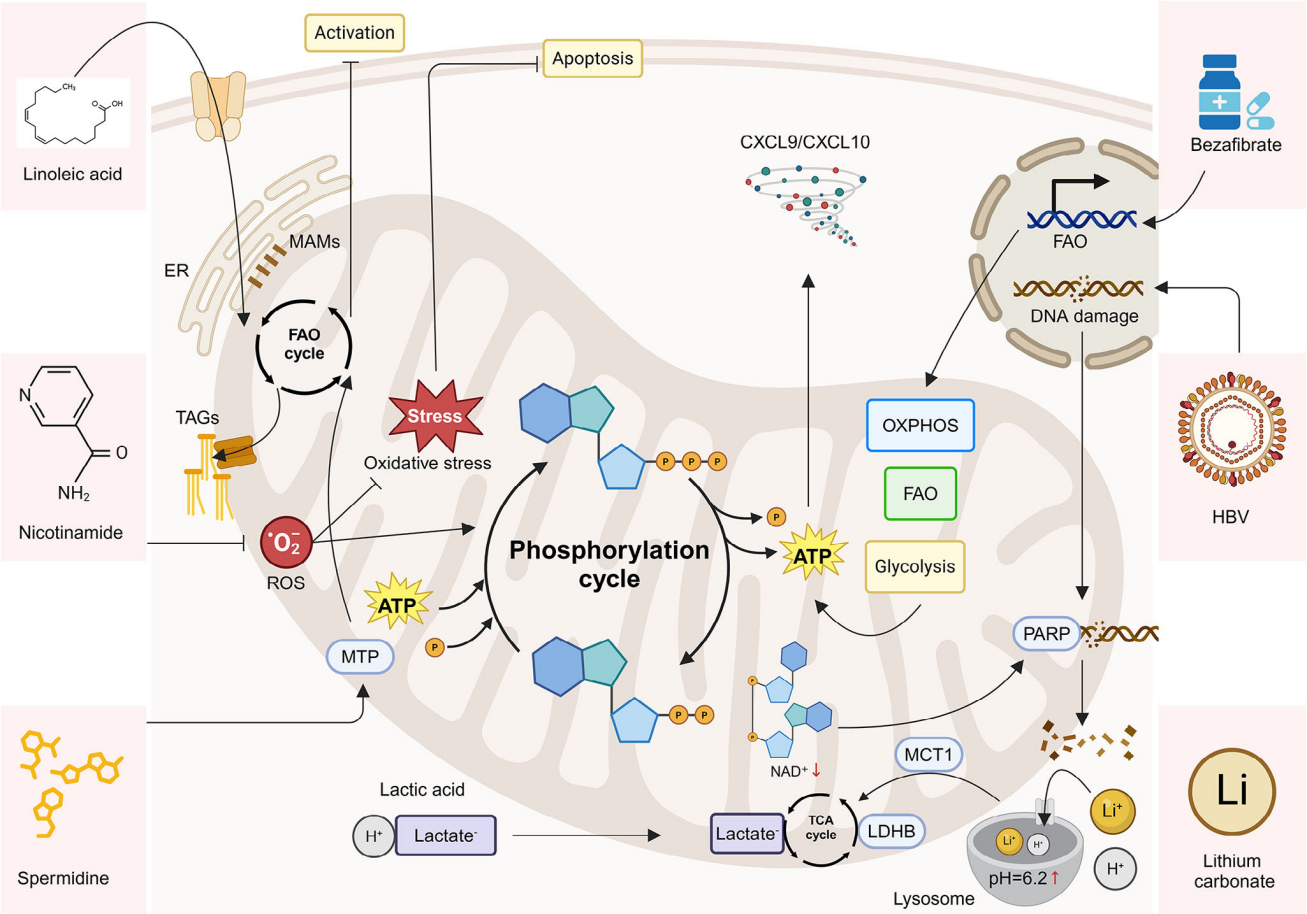
Mitochondrial modulation mechanisms in CD8⁺ T cells and their implications for enhancing immunotherapy efficacy. Key small molecules—linoleic acid, bezafibrate, nicotinamide (NAM), SPD, and lithium carbonate—boost mitochondrial function, metabolism, and T cell activation. Linoleic acid promotes mitochondrial energetics and calcium signaling, preventing T‐cell exhaustion. Bezafibrate enhances FAO and OXPHOS, improving T cell survival and activation. NAM reduces mitochondrial content and ROS, enhancing T‐cell proliferation. SPD activates MTP to support FAO, especially in aged T cells. Lithium carbonate rescues mitochondrial oxidation of LA, improving T cell function. Natural compounds like resveratrol and oleuropein also enhance mitochondrial activity in chronic HBV infection.

Engineered or synthetically designed components have also demonstrated efficacy in enhancing CD8^+^ T cell‐based immunotherapy. For instance, a half‐life‐extended interleukin‐10‐Fc fusion protein revitalizes terminally exhausted CD8⁺ T cells in tumors by enhancing oxidative phosphorylation, independent of progenitor cells. This metabolic reprogramming boosts T cell expansion and effector function, synergizing with adoptive T cell transfer to eradicate solid tumors and achieve durable cures in most treated mice.^[^ ^1^ ^]^ In addition, the DNA‐based epigenetic activator EnPGC‐1, targeting PGC‐1α/β, enhances mitochondrial biogenesis and oxidative phosphorylation in CD8⁺ T cells. This activation supports T cell proliferation, improves memory features, and when combined with PD‐1 blockade, significantly boosts antitumor immunity and survival, indicating a potent strategy for augmenting cancer immunotherapy.^[^ ^104^ ^]^ In addition to introducing exogenous substances to modulate mitochondrial function in CD8^+^ T cells, many novel strategies, such as the use of nanomaterials, temperature regulation, and genetic editing techniques, have also demonstrated promising results in enhancing T cell metabolism and improving immunotherapeutic outcomes. For instance, a novel magnetic metal‐organic framework nanoplatform coated with platelet membrane (PmMN@Om&As) enhances mitochondrial function in TILs by delivering oxymatrine and astragaloside IV directly into the hepatocellular carcinoma (HCC) microenvironment. This delivery system improves TIL activity, synergizes with PD‐1 inhibitors, and significantly suppresses tumor growth.^[^ ^105^ ^]^ Interestingly, exposure to febrile temperatures (39 °C) enhances CD8⁺ T cell metabolic activity and effector functions by upregulating mitochondrial pathways, increasing mitochondrial mass and metabolism. This temperature‐induced boost in mitochondrial translation improves the therapeutic efficacy of T cells in a myeloid leukemia model, suggesting that febrile temperature exposure could be harnessed to enhance T cell‐based immunotherapies.^[^ ^106^ ^]^ In addition, combinatorial anti‐PD‐L1/cryoablation therapy enhances mitochondrial function in PD‐1⁺CD8⁺ T cells, leading to improved IFN‐γ production and antitumor activity in a lung cancer model. This therapy specifically targets and revitalizes exhausted PD‐1⁺CD8⁺ T cells by correcting mitochondrial depolarization, underscoring the potential of targeting mitochondrial metabolism to augment CD8⁺ T cell efficacy in cancer treatment.^[^ ^107^ ^]^ Genetic engineering is effective in modulating mitochondrial function in CAR‐T cells. MCJ, a negative regulator of mitochondrial complex I, constrains mitochondrial respiration in CD8⁺ T cells, thereby limiting their anti‐tumor efficacy. Deletion or silencing of MCJ enhances mitochondrial metabolism, leading to an increase in vitro and in vivo activity of murine and human CD8⁺ CAR‐T cells against leukemia and melanoma.^[^ ^108^ ^]^ Similarly, the inclusion of 4‐1BB signaling domains in CAR T cell architecture promotes central memory CD8⁺ T cells with enhanced mitochondrial biogenesis and FAO, leading to improved respiratory capacity. Conversely, CAR T cells featuring CD28 domains favor effector memory cells with increased glycolysis. These metabolic differences potentially explain the varying persistence and exhaustion resistance observed in CAR‐T cells with different co‐stimulatory domains in clinical settings, guiding the design of more effective CAR T cell therapies (**Figure** **5** and **Table** **2**).^[^ ^109^ ^]^ Despite the breadth of strategies explored—ranging from small molecules and natural compounds to genetic and nanomaterial‐based interventions—several challenges limit their immediate translational potential. Many studies rely on murine models or in vitro assays that do not fully recapitulate the complexity of human T cell exhaustion or the TME. The context‐dependent nature of T cell metabolism, influenced by differentiation state, tissue localization, and disease setting, necessitates a more precise characterization of how these mitochondrial interventions affect specific CD8⁺ T cell subsets. Furthermore, the pleiotropic effects of metabolic modulators, especially in systemic delivery, raise concerns about unintended immunopathology or off‐target toxicity. Most critically, few approaches rigorously define therapeutic windows, the durability of mitochondrial reprogramming, or synergy with existing immunotherapies in clinically relevant settings. To realize their full therapeutic potential, robust pharmacokinetic profiling, longitudinal immune monitoring, and integration with emerging technologies—such as single‐cell metabolic imaging or spatial transcriptomics—will be essential to refine these strategies into targeted, safe, and durable adjuncts to immunotherapy.

**Table 2:** Mitochondrial modulation strategies and clinical outcomes in CD8⁺ T cell immunotherapies.

Strategies	Effect on CD8^+^ T Cell	Mechanism of Action	Clinical Implication	Refs.
LA treatment	Enhancing CD8^+^ T cell metabolic fitness	Enhancing ER‐mitochondria contacts and promoting Ca^2+^ signaling	Enhancing adoptive T cell therapy on myeloma and lymphoma	[93]
Bezafibrate treatment	Expanding infiltrating CD8^+^ T cells	Enhancing FAO in tumor‐infiltrating CTLs by upregulating FAO‐related genes	Adjuvant for PD‐1 blockade therapy within lung cancer	[94]
High‐dose NAM treatment	Enhancing CD8^+^ T cell expansion	Inhibiting ROS generation and reducing mitochondrial content	For the treatment of immune deficiency or autoimmune diseases	[96]
NAD supplementation and CD38 inhibition	Restoring HBV‐specific CD8^+^ T cell function	Improving DNA repair mechanisms, mitochondrial function, and proteostasis	Therapy for chronic HBV infection	[98]
SPD treatment	Enhancing CD8^+^ T cell antitumor immunity	Enhancing MTP enzymatic activity and increasing FAO	Improving age‐related decline in immune function	[99]
LC treatment	Promoting CD8^+^ T cell activation	Promoting MCT1 localization to mitochondrial membranes	Enhancing Colon Cancer Immunotherapy	[100]
RSV and OLE treatment	Correcting intracellular defects in CD8^+^ T cell	Enhancing autophagic flux and reducing oxidative stress	Developing novel immune‐based therapies for HBV	[21]
Combination of Om and As treatment	Enhancing CD8^+^ T cell infiltration	Suppressing CAF activation and promoting T cell mitochondrial activity	For TNBC treatment	[101]
Berberine treatment	Inducing T cell apoptosis	Reducing CD4^+^ and CD8^+^ T cell infiltration and inhibiting T cell activation	Enhancing allograft survival	[102]
YQJPF treatment	Enhancing CD8^+^ T cell autophagy	Upregulating mitochondrial biogenesis in CD8^+^ T cells by enhancing PGC‐1α, NRF‐1, and TFAM expression	Improving Immune Therapy in ACLF	[103]
EnPGC‐1 treatment	Targeting epigenetic induction in CD8^+^ T cell	Targeting PGC‐1α/β and promoting oxidative phosphorylation	Treating OXPHOS‐related mitochondrial disorders	[104]
PmMN@Om&As treatment	Co‐delivering drugs to modulate TILs	Inhibiting CAFs and enhancing TILs' mitochondrial function	Improving antitumor effect of immunotherapy in HCC	[105]
Exposure to febrile temperature	Enhancing CD8^+^ T cell metabolic activity	Upregulating mitochondrial pathways, increasing mitochondrial mass	Enhancing T cell‐based therapies of myeloid leukemia and melanoma	[106]
Anti‐PD‐L1 and cryoablation therapy	Modulating mitochondrial metabolism in CD8^+^ T cell	Improving mitochondrial metabolism and IFN‐γ production in PD‐1+CD8^+^ T cells	Improving Prognosis in lung cancer	[107]
Inhibiting MCJ	Enhancing CD8^+^ T cell adoptive	Increasing mitochondrial respiration and metabolism	Improving adoptive T cell therapies for leukemia	[108]
CD28 and 4‐1BB treatment	Enhancing CAR T cell memory development	Enhancing respiratory capacity, FAO, and mitochondrial biogenesis	Improving treatment of CLL and other hematologic malignancies	[109]

## 6 Challenges and Future Perspectives

Mitochondrial modulation represents a highly promising therapeutic avenue for enhancing CD8⁺ T cell immunity.^[^ ^79^ ^]^ Mitochondria‐targeted system therapy combined with radiofrequency ablation offers a potential alternative to surgery for early‐stage non‐small cell lung cancer. Preliminary clinical data suggest this approach is safe and effective over short‐term follow‐up (NCT03840408). However, significant practical challenges must be systematically addressed before broad clinical translation can be achieved. Herein, we discuss critical open questions, elaborate feasible solutions, and highlight specific approaches currently under investigation.

**Figure 6:**
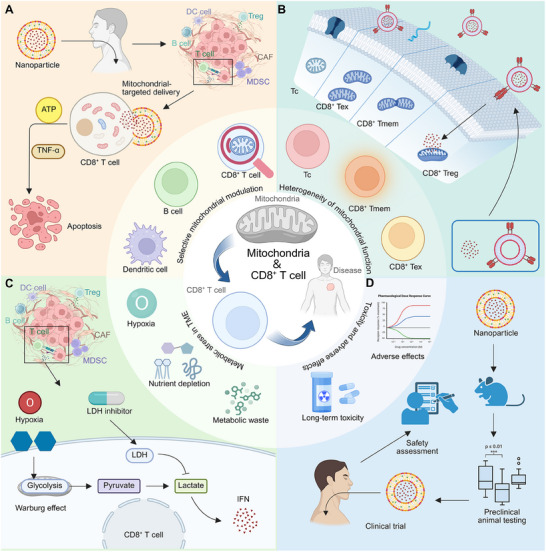
Mitochondrial regulation of CD8⁺ T cell function and therapeutic interventions. A) Mitochondria‐targeted nanoparticle delivery modulates CD8⁺ T cell metabolism in the TME. These nanoparticles influence ATP production and TNF‐α signaling, potentially inducing CD8⁺ T cell apoptosis. Interactions between CD8⁺ T cells, DCs, Tregs, CAFs, and MDSCs shape mitochondrial responses. B) Heterogeneity in CD8⁺ T cell mitochondrial function drives functional diversity, including cytotoxic T cells, memory T cells, and exhausted T cells. Mitochondrial modulation strategies can influence Treg differentiation, with potential implications for immunotherapies. C) Hypoxia and metabolic constraints in the TME shift CD8⁺ T cell metabolism toward glycolysis, reinforcing the Warburg effect. LDH‐mediated lactate production under hypoxic conditions suppresses IFN responses, which can be counteracted using LDH inhibitors to restore mitochondrial function. D) Nanoparticle‐based immunotherapies require safety evaluation to assess pharmacological response, adverse effects, and long‐term toxicity. Preclinical animal testing and clinical trials are essential for validating therapeutic efficacy and safety.

A primary challenge in mitochondrial‐targeted therapies is achieving highly selective modulation of mitochondrial pathways specifically within CD8⁺ T cells. Non‐specific modulation poses significant risks, as inadvertent alteration of mitochondrial function in other immune or non‐immune cell populations could potentially cause systemic side effects and off‐target toxicity. To address this critical limitation, strategies employing targeted nanoparticle‐based drug delivery systems, liposomes, or antibody‐directed ligands designed to bind specifically to CD8⁺ T cell surface markers, such as CD8a or CD8b, have emerged as feasible solutions. Recent advancements in nanotechnology and ligand engineering have significantly enhanced the precision, specificity, and overall efficiency of these delivery platforms. A notable example of this approach is the magnetic metal‐organic framework nanoplatform coated with platelet membranes (PmMN@Om&As), which selectively delivers oxymatrine and astragaloside IV specifically to HCC tissues. This strategy exemplifies the efficacy of targeted mitochondrial modulation, as it effectively regulates cancer‐associated fibroblasts and enhances mitochondrial function in TILs, substantially boosting TIL activity and synergizing effectively with PD‐1 blockade therapy to achieve significant antitumor efficacy.^[^ ^105^ ^]^ Complementing these targeted delivery systems, gene‐editing technologies such as CRISPR/Cas9 offer additional compelling strategies for the precise manipulation of mitochondrial function within CD8⁺ T cells. CRISPR/Cas9 can be used to directly alter genes that control mitochondrial biogenesis, mitochondrial dynamics, or key metabolic regulators. However, despite its potential, clinical translation of gene editing faces several critical barriers, including high development and manufacturing costs, regulatory complexity, potential off‐target genetic effects, ethical considerations, and variable editing efficiency. Nonetheless, the growing number of ongoing clinical trials utilizing CRISPR‐based genetic editing in T cells‐such as gene‐edited PD‐1 knockout T cells or CRISPR‐engineered CAR T‐cell therapies‐demonstrate its increasing feasibility in clinical settings.^[^ ^110^ ^]^ To facilitate broader clinical adoption, careful preclinical optimization, enhanced genome‐editing specificity via base editing or prime editing techniques, improvements in delivery vectors such as non‐viral lipid nanoparticles or electroporation systems, and robust safety monitoring frameworks will be essential. Furthermore, comprehensive cost‐benefit analyses comparing CRISPR‐based mitochondrial editing strategies to current standard therapies are critical to clarify the long‐term feasibility, practicality, and affordability of these innovative approaches in routine clinical practice (**Figure** **6**A). T cells, including CD8⁺ subsets, exhibit marked heterogeneity in mitochondrial function, which varies according to their activation status, differentiation trajectory, and tissue localization. Distinct T cell populations—such as naïve, effector, memory, and exhausted subsets‐ harbor unique mitochondrial profiles defined by differences in mitochondrial mass, membrane potential, spare respiratory capacity, and metabolic plasticity.^[^ ^111^ ^]^ This functional diversity presents a major challenge to the development of generalized mitochondrial‐targeted strategies. For instance, interventions that promote mitochondrial biogenesis may enhance the function of exhausted CD8⁺ T cells in tumors or chronic infections, yet could concurrently amplify the activation and survival of autoreactive effector T cells in autoimmune settings, thereby exacerbating immunopathology.^[^ ^112^ ^]^ One open question is how to tailor mitochondrial interventions to subset‐specific demands without triggering undesirable immune activation elsewhere. Addressing this requires systematic mitochondrial profiling of T‐cell subsets under both homeostatic and pathological conditions. Single‐cell transcriptomics, mitochondrial proteomics, and high‐resolution metabolic imaging offer powerful tools to dissect these subset‐specific signatures. In addition, integrating metabolic features into T‐cell classification frameworks may enable the development of mitochondria‐targeted approaches that selectively modulate defined T‐cell populations based on their unique bioenergetic needs, differentiation stage, or tissue microenvironment (Figure 6B).

In the context of cancer immunotherapy, the TME imposes profound metabolic constraints on infiltrating immune cells, including CD8⁺ T cells. Hypoxia, glucose and amino acid deprivation, excess lipid accumulation, and the buildup of metabolic waste products such as lactate collectively disrupt mitochondrial integrity and impair oxidative metabolism, ultimately promoting T‐cell dysfunction and exhaustion.^[^ ^113^ ^]^ Overcoming these environmental constraints is essential for restoring mitochondrial function in T cells and sustaining antitumor activity. A major unresolved question is how to decouple T cell‐intrinsic mitochondrial repair from extrinsic TME‐induced metabolic suppression. One promising solution involves the co‐targeting of mitochondrial metabolism in CD8⁺ T cells alongside metabolic remodeling of the TME. For example, LDH inhibitors, MCT1/4 blockade, or lactate‐scavenging nanoparticles can alleviate lactic acid‐induced mitochondrial stress. Additionally, vascular normalization agents or local oxygen delivery platforms may reduce hypoxia and enhance mitochondrial respiration. Combinatorial strategies that simultaneously modulate both T cell metabolism and environmental constraints—while preserving selectivity and minimizing toxicity—represent a compelling path forward. Engineering CAR T cells with mitochondria‐stabilizing factors or metabolic enhancers may further improve persistence and cytotoxicity under metabolically hostile conditions (Figure 6C).

Beyond context‐specific challenges, a fundamental concern in mitochondria‐targeted therapies is the potential for long‐term toxicity and unintended consequences due to the central role of mitochondria in core cellular functions. As mitochondria are involved in ATP production, apoptosis regulation, redox homeostasis, and calcium buffering, even mild dysregulation can propagate cellular stress and organ dysfunction.^[^ ^114^ ^]^ An important open question is how to balance therapeutic efficacy with safety when modulating such a universally essential organelle. Longitudinal safety assessments must include not only acute toxicity profiles but also evaluations of mitochondrial DNA stability, cumulative oxidative stress, and tissue‐specific mitochondrial reserve capacity. To mitigate risks, precision delivery systems that target mitochondrial modulators specifically to CD8⁺ T cells—or even to subpopulations of interest—are under development. Nanocarriers conjugated to CD8‐specific ligands, tissue‐penetrating peptides, or immune checkpoint‐targeting antibodies can help restrict mitochondrial modulation to disease‐relevant immune compartments. Moreover, designing therapies with tunable, reversible, or dose‐dependent effects‐such as small molecule modulators with rapid clearance, or light‐ or ligand‐inducible gene expression systems offer temporal control to prevent overactivation or irreversible mitochondrial injury. Collectively, these strategies aim to ensure that mitochondria‐targeted immunotherapies are not only effective but also sustainable and safe for long‐term use in the treatment of cancer, infection, and autoimmune disease (Figure 6D).

## 7 Conclusion

Mitochondrial function plays a pivotal role in the efficacy of CD8^+^ T cells during immune responses, influencing their activation, differentiation, and persistence. Mitochondrial dysfunction in CD8^+^ T cells contributes to immune exhaustion, impairing both antitumor and antiviral immunity. This underscores the necessity for targeted strategies to restore mitochondrial integrity. Key mitochondrial processes—such as biogenesis, metabolism, redox balance, and dynamics—are essential for optimizing CD8^+^ T cell‐based immunotherapy. Current strategies aimed at enhancing mitochondrial function, including metabolic reprogramming and mitochondrial‐targeted therapies, hold promise for therapeutic intervention. However, challenges remain, particularly in achieving selective targeting, managing mitochondrial plasticity, and addressing potential off‐target effects. Future research should focus on refining techniques for mitochondrial modulation, integrating them with immune checkpoint blockade and other immunotherapies, and ensuring their safety and efficacy in clinical applications. By advancing our understanding of mitochondrial dynamics in CD8^+^ T cells, there is significant potential to enhance immune‐based therapies and restore immune homeostasis in various disease contexts.

## Conflict of Interest

The authors declare no competing interests.

## Author Contributions

X.Z. and L.C. conceived of the presented idea. X.Z., L.C., X.C., J.Z., Y.L., Y.F.L., P.L., and Z.H.Z. wrote the manuscript. X.C. created the graphs and figures. All authors contributed to and approved the final manuscript.

## References

[1] Y. Guo , Y. Q. Xie , M. Gao , Y. Zhao , F. Franco , M. Wenes , I. Siddiqui , A. Bevilacqua , H. Wang , H. Yang , B. Feng , X. Xie , C. M. Sabatel , B. Tschumi , A. Chaiboonchoe , Y. Wang , W. Li , W. Xiao , W. Held , P. Romero , P. C. Ho , L. Tang , Nat. Immunol. 2021, 22, 746. 34031618 10.1038/s41590-021-00940-2 PMC7610876

[2] A. Angajala , S. Lim , J. B. Phillips , J. H. Kim , C. Yates , Z. You , M. Tan , Front. Immunol. 2018, 9, 1605. 30050539 10.3389/fimmu.2018.01605 PMC6052888

[3] P. J. Peters , J. Borst , V. Oorschot , M. Fukuda , O. Krähenbühl , J. Tschopp , J. W. Slot , H J. Geuze , J. Exp. Med. 1991, 173, 1099. 2022921 10.1084/jem.173.5.1099 PMC2118839

[4] J. Nunnari , A. Suomalainen , Cell 2012, 148, 1145. 22424226 10.1016/j.cell.2012.02.035 PMC5381524

[5] D. O'Sullivan , E L. Pearce , Trends Immunol. 2015, 36, 71. 25601541 10.1016/j.it.2014.12.004 PMC4323623

[6] C. Abad , I. Pinal‐Fernandez , C. Guillou , G. Bourdenet , L. Drouot , P. Cosette , M. Giannini , L. Debrut , L. Jean , S. Bernard , D. Genty , R. Zoubairi , I. Remy‐Jouet , B. Geny , C. Boitard , A. Mammen , A. Meyer , O. Boyer , Nat. Commun. 2024, 15, 5403. 38926363 10.1038/s41467-024-49460-1 PMC11208592

[7] A. Schurich , L. J. Pallett , D. Jajbhay , J. Wijngaarden , I. Otano , U. S. Gill , N. Hansi , P. T. Kennedy , E. Nastouli , R. Gilson , C. Frezza , S. M. Henson , M K. Maini , Cell Rep. 2016, 16, 1243. 27452473 10.1016/j.celrep.2016.06.078 PMC4977274

[8] K. E. Beckermann , R. Hongo , X. Ye , K. Young , K. Carbonell , D. C. C. Healey , P. J. Siska , S. Barone , C. E. Roe , C. C. Smith , B. G. Vincent , F. M. Mason , J. M. Irish , W. K. Rathmell , J C. Rathmell , JCI Insight 2020, 5. 10.1172/jci.insight.138729 PMC7455120 32814710

[9] R. J. Kishton , M. Sukumar , N P. Restifo , Cell Metab. 2017, 26, 94. 28683298 10.1016/j.cmet.2017.06.016 PMC5543711

[10] M. Liesa , M. Palacín , A. Zorzano , Physiol. Rev. 2009, 89, 799. 19584314 10.1152/physrev.00030.2008

[11] C. F. Lee , Y. C. Lo , C. H. Cheng , G. J. Furtmüller , B. Oh , V. Andrade‐Oliveira , A. G. Thomas , C. E. Bowman , B. S. Slusher , M. J. Wolfgang , G. Brandacher , J D. Powell , Cell Rep. 2015, 13, 760. 26489460 10.1016/j.celrep.2015.09.036 PMC4626381

[12] L. A. Sena , S. Li , A. Jairaman , M. Prakriya , T. Ezponda , D. A. Hildeman , C. R. Wang , P. T. Schumacker , J. D. Licht , H. Perlman , P. J. Bryce , Immunity 2013, 38, 225. 23415911 10.1016/j.immuni.2012.10.020 PMC3582741

[13] A. Teijeira , S. Labiano , S. Garasa , I. Etxeberria , E. Santamaría , A. Rouzaut , M. Enamorado , A. Azpilikueta , S. Inoges , E. Bolaños , M. A. Aznar , A. R. Sánchez‐Paulete , D. Sancho , I. Melero , Cancer Immunol. Res. 2018, 6, 798. 29678874 10.1158/2326-6066.CIR-17-0767

[14] K. Tang , H. Zhang , J. Deng , D. Wang , S. Liu , S. Lu , Q. Cui , C. Chen , J. Liu , Z. Yang , Y. Li , J. Chen , J. Lv , J. Ma , B. Huang , Nat. Immunol. 2023, 24, 162. 36471170 10.1038/s41590-022-01365-1

[15] P. Khurana , C. Burudpakdee , S. A. Grupp , U. H. Beier , D. M. Barrett , H. Bassiri , Front. Immunol. 2021, 12, 700374. 34434191 10.3389/fimmu.2021.700374 PMC8380770

[16] W. Wang , M. N. Guo , N. Li , D. Q. Pang , J H. Wu , World J. Gastrointest. Oncol. 2022, 14, 1124. 35949216 10.4251/wjgo.v14.i6.1124 PMC9244988

[17] G. J. van der Windt , B. Everts , C. H. Chang , J. D. Curtis , T. C. Freitas , E. Amiel , E. J. Pearce , E. L. Pearce , Immunity 2012, 36, 68. 22206904 10.1016/j.immuni.2011.12.007 PMC3269311

[18] M. D. Buck , D. O'Sullivan , R. I. Klein Geltink , J. D. Curtis , C. H. Chang , D. E. Sanin , J. Qiu , O. Kretz , D. Braas , G. J. van der Windt , Q. Chen , S. C. Huang , C. M. O'Neill , B. T. Edelson , E. J. Pearce , H. Sesaki , T. B. Huber , A. S. Rambold , E. L. Pearce , Cell 2016, 166, 63. 27293185 10.1016/j.cell.2016.05.035 PMC4974356

[19] Y. N. Liu , J. F. Yang , D. J. Huang , H. H. Ni , C. X. Zhang , L. Zhang , J. He , J. M. Gu , H. X. Chen , H. Q. Mai , Q. Y. Chen , X. S. Zhang , S. Gao , J. Li , Front. Immunol. 2020, 11, 1906. 32973789 10.3389/fimmu.2020.01906 PMC7472844

[20] A. Alrubayyi , E. Moreno‐Cubero , D. Hameiri‐Bowen , R. Matthews , S. Rowland‐Jones , A. Schurich , D. Peppa , Front. Immunol. 2022, 13, 908697. 35865519 10.3389/fimmu.2022.908697 PMC9295450

[21] G. Acerbi , I. Montali , G. D. Ferrigno , V. Barili , S. Schivazappa , A. Alfieri , D. Laccabue , A. Loglio , M. Borghi , M. Massari , M. Rossi , A. Vecchi , A. Penna , C. Boni , G. Missale , P. Lampertico , D. Del Rio , C. Ferrari , P. Fisicaro , J. Hepatol. 2021, 74, 783. 33188902 10.1016/j.jhep.2020.10.034

[22] Y. Mo , K. K. To , R. Zhou , L. Liu , T. Cao , H. Huang , Z. Du , C. Y. H. Lim , L. Y. Yim , T. Y. Luk , J. M. Chan , T. S. Chik , D. P. Lau , O. T. Tsang , A. R. Tam , I. F. Hung , K. Y. Yuen , Z. Chen , Front. Immunol. 2021, 12, 799896. 35095881 10.3389/fimmu.2021.799896 PMC8795605

[23] S. L. Russell , D. A. Lamprecht , T. Mandizvo , T. T. Jones , V. Naidoo , K. W. Addicott , C. Moodley , B. Ngcobo , D. K. Crossman , G. Wells , A J C. Steyn , Cell Rep. 2019, 29, 3564. 31825836 10.1016/j.celrep.2019.11.034 PMC6915325

[24] P. M. Chen , E. Katsuyama , A. Satyam , H. Li , J. Rubio , S. Jung , S. Andrzejewski , J. D. Becherer , M. G. Tsokos , R. Abdi , G C. Tsokos , Sci. Adv. 2022, 8, 4271. 10.1126/sciadv.abo4271 PMC9200274 35704572

[25] N. Buang , L. Tapeng , V. Gray , A. Sardini , C. Whilding , L. Lightstone , T. D. Cairns , M. C. Pickering , J. Behmoaras , G. S. Ling , M. Botto , Nat. Commun. 2021, 12, 1980. 33790300 10.1038/s41467-021-22312-y PMC8012390

[26] D. Luo , L. Li , Y. Wu , Y. Yang , Y. Ye , J. Hu , Y. Gao , N. Zeng , X. Fei , N. Li , L. Jiang , Front. Immunol. 2023, 14, 1156774. 37497211 10.3389/fimmu.2023.1156774 PMC10366690

[27] A. V. Menk , N. E. Scharping , D. B. Rivadeneira , M. J. Calderon , M. J. Watson , D. Dunstane , S. C. Watkins , G M. Delgoffe , J. Exp. Med. 2018, 215, 1091. 29511066 10.1084/jem.20171068 PMC5881463

[28] V. Verma , N. Jafarzadeh , S. Boi , S. Kundu , Z. Jiang , Y. Fan , J. Lopez , R. Nandre , P. Zeng , F. Alolaqi , S. Ahmad , P. Gaur , S. T. Barry , V.‐A. VE , P. D. Smith , J. Banchereau , M. Mkrtichyan , B. Youngblood , P. C. Rodriguez , S. Gupta , S N. Khleif , Nat. Immunol. 2021, 22, 53. 33230330 10.1038/s41590-020-00818-9 PMC10081014

[29] L. A. Callender , E. C. Carroll , E. A. Bober , A. N. Akbar , E. Solito , S M. Henson , Aging Cell 2020, 19, 13067. 10.1111/acel.13067 PMC6996952 31788930

[30] G. J. van der Windt , D. O'Sullivan , B. Everts , S. C. Huang , M. D. Buck , J. D. Curtis , C. H. Chang , A. M. Smith , T. Ai , B. Faubert , R. G. Jones , E. J. Pearce , E. L. Pearce , Proc. Natl. Acad. Sci. U.S.A. 2013, 110, 14336. 23940348 10.1073/pnas.1221740110 PMC3761631

[31] K. Chamoto , P. S. Chowdhury , A. Kumar , K. Sonomura , F. Matsuda , S. Fagarasan , T. Honjo , Proc. Natl. Acad. Sci. U.S.A. 2017, 114, 761. 10.1073/pnas.1620433114 PMC5293087 28096382

[32] O. Abu Shelbayeh , T. Arroum , S. Morris , K. B. Busch , Antioxidants 2023, 12. 10.3390/antiox12051075 PMC10215733 37237941

[33] M. Fischer , G. R. Bantug , S. Dimeloe , P. M. Gubser , A. V. Burgener , J. Grählert , M. L. Balmer , L. Develioglu , R. Steiner , G. Unterstab , U. Sauder , G. Hoenger , C. Hess , Eur. J. Immunol. 2018, 48, 1632. 30028501 10.1002/eji.201747443

[34] X. Zhong , H. Wu , C. Ouyang , W. Zhang , Y. Shi , Y. C. Wang , D. K. Ann , Y. Gwack , W. Shang , Z. Sun , Cancer Immunol. Res. 2023, 11, 1414. 37540802 10.1158/2326-6066.CIR-23-0092 PMC10592187

[35] J. A. C. van Bruggen , A. W. J. Martens , J. A. Fraietta , T. Hofland , S. H. Tonino , E. Eldering , M. D. Levin , P. J. Siska , S. Endstra , J. C. Rathmell , C. H. June , D. L. Porter , J. J. Melenhorst , A. P. Kater , G J W. van der Windt , Blood 2019, 134, 44. 31076448 10.1182/blood.2018885863 PMC7022375

[36] A. Radziszewska , H. Peckham , R. Restuadi , M. Kartawinata , D. Moulding , N. M. de Gruijter , G. A. Robinson , M. Butt , C. T. Deakin , M. G. L. Wilkinson , L. R. Wedderburn , E. C. Jury , E. C. Rosser , C. Ciurtin , Clin. Exp. Immunol. 2025, 219, 127. 10.1093/cei/uxae127 PMC11748002 39719886

[37] T. Chao , H. Wang , P. C. Ho , Front. Immunol. 2017, 8, 473. 28484465 10.3389/fimmu.2017.00473 PMC5401871

[38] M. Corrado , D. Samardžić , M. Giacomello , N. Rana , E. L. Pearce , L. Scorrano , Cell Death Differ. 2021, 28, 2194. 33649469 10.1038/s41418-021-00747-6 PMC8257785

[39] J. He , X. Shangguan , W. Zhou , Y. Cao , Q. Zheng , J. Tu , G. Hu , Z. Liang , C. Jiang , L. Deng , S. Wang , W. Yang , Y. Zuo , J. Ma , R. Cai , Y. Chen , Q. Fan , B. Dong , W. Xue , H. Tan , Y. Qi , J. Gu , B. Su , Y. Eugene Chin , G. Chen , Q. Wang , T. Wang , J. Cheng , Nat. Commun. 2021, 12, 4371. 34272364 10.1038/s41467-021-24619-2 PMC8285428

[40] L. Simula , Y. Antonucci , G. Scarpelli , V. Cancila , A. Colamatteo , S. Manni , B. De Angelis , C. Quintarelli , C. Procaccini , G. Matarese , C. Tripodo , S. Campello , Mol. Oncol. 2022, 16, 188. 34535949 10.1002/1878-0261.13103 PMC8732338

[41] Y. Zhang , W. Li , K. Ma , J. Zhai , Y. Jin , L. Zhang , C. Chen , Immunol. Lett. 2022, 245, 61. 35429545 10.1016/j.imlet.2022.04.003

[42] H. Wu , X. Zhao , S. M. Hochrein , M. Eckstein , G. F. Gubert , K. Knöpper , A. M. Mansilla , A. Öner , R. Doucet‐Ladevèze , W. Schmitz , B. Ghesquière , S. Theurich , J. Dudek , G. Gasteiger , A. Zernecke , S. Kobold , W. Kastenmüller , M. Vaeth , Nat. Commun. 2023, 14, 6858. 37891230 10.1038/s41467-023-42634-3 PMC10611730

[43] N. E. Scharping , D. B. Rivadeneira , A. V. Menk , P. D. A. Vignali , B. R. Ford , N. L. Rittenhouse , R. Peralta , Y. Wang , Y. Wang , K. DePeaux , A. C. Poholek , G M. Delgoffe , Nat. Immunol. 2021, 22, 205. 33398183 10.1038/s41590-020-00834-9 PMC7971090

[44] S. A. Vardhana , M. A. Hwee , M. Berisa , D. K. Wells , K. E. Yost , B. King , M. Smith , P. S. Herrera , H. Y. Chang , A. T. Satpathy , M. R. M. van den Brink , J. R. Cross , C. B. Thompson , Nat. Immunol. 2020, 21, 1022. 32661364 10.1038/s41590-020-0725-2 PMC7442749

[45] C. M. Moshfegh , C. W. Collins , V. Gunda , A. Vasanthakumar , J. Z. Cao , P. K. Singh , L. A. Godley , A J. Case , Redox Biol. 2019, 27, 101141. 30819616 10.1016/j.redox.2019.101141 PMC6859572

[46] P. J. Siska , K. E. Beckermann , F. M. Mason , G. Andrejeva , A. R. Greenplate , A. B. Sendor , Y. J. Chiang , A. L. Corona , L. F. Gemta , B. G. Vincent , R. C. Wang , B. Kim , J. Hong , C. L. Chen , T. N. Bullock , J. M. Irish , W. K. Rathmell , J C. Rathmell , JCI Insight 2017, 2. 10.1172/jci.insight.93411 PMC5470888 28614802

[47] M. Liu , X. Fu , Q. Yi , E. Xu , L. Dong , Biochem. Biophys. Res. Commun. 2024, 734, 150738. 39342799 10.1016/j.bbrc.2024.150738

[48] A. K. Grotle , A. M. Darling , E. F. Saunders , P. J. Fadel , D. W. Trott , J L. Greaney , Am. J. Physiol. Heart Circ. Physiol. 2022, 322, 568. 10.1152/ajpheart.00019.2022 PMC8917910 35179977

[49] S. Y. Li , L. B. Yin , H. B. Ding , M. Liu , J. N. Lv , J. Q. Li , J. Wang , T. Tang , Y. J. Fu , Y. J. Jiang , Z. N. Zhang , H. Shang , Front. Immunol. 2023, 14, 1106881. 36875092 10.3389/fimmu.2023.1106881 PMC9981933

[50] M. Hoth , D. C. Button , R S. Lewis , Proc. Natl. Acad. Sci. U.S.A. 2000, 97, 10607. 10973476 10.1073/pnas.180143997 PMC27072

[51] A. Quintana , M. Pasche , C. Junker , D. Al‐Ansary , H. Rieger , C. Kummerow , L. Nuñez , C. Villalobos , P. Meraner , U. Becherer , J. Rettig , B. A. Niemeyer , M. Hoth , EMBO J. 2011, 30, 3895. 21847095 10.1038/emboj.2011.289 PMC3209779

[52] J. F. Yang , X. Xing , L. Luo , X. W. Zhou , J. X. Feng , K. B. Huang , H. Liu , S. Jin , Y. N. Liu , S. H. Zhang , Y. H. Pan , B. Yu , J. Y. Yang , Y. L. Cao , Y. Cao , C. Y. Yang , Y. Wang , Y. Zhang , J. Li , X. Xia , T. Kang , R. H. Xu , P. Lan , J. H. Luo , H. Han , F. Bai , S. Gao , Sci. Immunol. 2023, 8, 2424. 10.1126/sciimmunol.abq2424 37738362

[53] C. Ding , Y. Hong , Y. Che , T. He , Y. Wang , S. Zhang , J. Wu , W. Xu , J. Hou , H. Hao , L. Cao , FASEB J. 2022, 36, 22468. 10.1096/fj.202200332R 35913801

[54] M. Khattar , Y. Miyahara , P. M. Schroder , A. Xie , W. Chen , S M. Stepkowski , PLoS One 2014, 9, 85882. 10.1371/journal.pone.0085882 PMC3887105 24416451

[55] J. D. Tejero , R. S. Hesterberg , S. Drapela , D. Ilter , D. Raizada , F. Lazure , H. Kashfi , M. Liu , L. Silvane , D. Avram , J. Fernández‐García , J. M. Asara , S. M. Fendt , J. L. Cleveland , A P. Gomes , Oncogene 2025, 44, 105. 39472497 10.1038/s41388-024-03191-1 PMC12904991

[56] K. Renner , A. L. Geiselhöringer , M. Fante , C. Bruss , S. Färber , G. Schönhammer , K. Peter , K. Singer , R. Andreesen , P. Hoffmann , P. Oefner , W. Herr , M. Kreutz , Eur. J. Immunol. 2015, 45, 2504. 26114249 10.1002/eji.201545473

[57] A. V. Menk , N. E. Scharping , R. S. Moreci , X. Zeng , C. Guy , S. Salvatore , H. Bae , J. Xie , H. A. Young , S. G. Wendell , G M. Delgoffe , Cell Rep. 2018, 22, 1509. 29425506 10.1016/j.celrep.2018.01.040 PMC5973810

[58] M. Wenes , A. Jaccard , T. Wyss , N. Maldonado‐Pérez , S. T. Teoh , A. Lepez , F. Renaud , F. Franco , P. Waridel , C. Yacoub Maroun , B. Tschumi , N. Dumauthioz , L. Zhang , A. Donda , F. Martín , D. Migliorini , S. Y. Lunt , P. C. Ho , P. Romero , Cell Metab. 2022, 34, 731. 35452600 10.1016/j.cmet.2022.03.013 PMC9116152

[59] Y. Elbaz , M. Schuldiner , Trends Biochem. Sci. 2011, 36, 616. 21958688 10.1016/j.tibs.2011.08.004

[60] G. R. Bantug , M. Fischer , J. Grählert , M. L. Balmer , G. Unterstab , L. Develioglu , R. Steiner , L. Zhang , A. S. H. Costa , P. M. Gubser , A. V. Burgener , U. Sauder , J. Löliger , R. Belle , S. Dimeloe , J. Lötscher , A. Jauch , M. Recher , G. Hönger , M. N. Hall , P. Romero , C. Frezza , C. Hess , Immunity 2018, 48, 542. 29523440 10.1016/j.immuni.2018.02.012 PMC6049611

[61] S. Ma , L. T. Ong , Z. Jiang , W. C. Lee , P. L. Lee , M. Yusuf , H. J. Ditzel , Y. Wang , Q. Chen , W. Wang , X. Wu , E. Y. Tan , Q. Yu , Cancer Cell 2025, 43, 213. 39729997 10.1016/j.ccell.2024.12.001

[62] L. F. Gemta , P. J. Siska , M. E. Nelson , X. Gao , X. Liu , J. W. Locasale , H. Yagita , C. L. Slingluff Jr. , K. L. Hoehn , J. C. Rathmell , T. N. J. Bullock , Sci. Immunol. 2019, 4. 10.1126/sciimmunol.aap9520 PMC6824424 30683669

[63] Y. Sun , N. K. Preiss , K. B. Valenteros , Y. Kamal , Y. K. Usherwood , H. R. Frost , E J. Usherwood , J. Immunol. 2020, 205, 2649. 32998985 10.4049/jimmunol.2000459 PMC7931848

[64] J. Li , Y. L. Yang , L. Z. Li , L. Zhang , Q. Liu , K. Liu , P. Li , B. Liu , L W. Qi , Biochim. Biophys. Acta. Mol. Basis Dis. 2017, 1863, 2835. 28736181 10.1016/j.bbadis.2017.07.017

[65] C. Chen , H. Zheng , E. M. Horwitz , S. Ando , K. Araki , P. Zhao , Z. Li , M. L. Ford , R. Ahmed , C K. Qu , Sci. Adv. 2023, 9, 9522. 10.1126/sciadv.adf9522 PMC10699783 38055827

[66] H. Guo , Z. Hu , X. Yang , Z. Yuan , Y. Gao , J. Chen , L. Xie , C. Chen , Y. Guo , Y. Bai , Int. Immunopharmacol. 2023, 123, 110709. 37515849 10.1016/j.intimp.2023.110709

[67] T. Manzo , B. M. Prentice , K. G. Anderson , A. Raman , A. Schalck , G. S. Codreanu , C. B. Nava Lauson , S. Tiberti , A. Raimondi , M. A. Jones , M. Reyzer , B. M. Bates , J. M. Spraggins , N. H. Patterson , J. A. McLean , K. Rai , C. Tacchetti , S. Tucci , J. A. Wargo , S. Rodighiero , K. Clise‐Dwyer , S. D. Sherrod , M. Kim , N. E. Navin , R. M. Caprioli , P. D. Greenberg , G. Draetta , L. Nezi , J. Exp. Med. 2020, 217. 10.1084/jem.20191920 PMC7398173 32491160

[68] C. Ledderose , Y. Bao , M. Lidicky , J. Zipperle , L. Li , K. Strasser , N. I. Shapiro , W G. Junger , J. Biol. Chem. 2014, 289, 25936. 25070895 10.1074/jbc.M114.575308 PMC4162192

[69] A. M. Amitrano , B. J. Berry , K. Lim , K. D. Kim , R. E. Waugh , A. P. Wojtovich , M. Kim , Front. Immunol. 2021, 12, 666231. 34149701 10.3389/fimmu.2021.666231 PMC8209468

[70] S. Wang , H. Long , L. Hou , B. Feng , Z. Ma , Y. Wu , Y. Zeng , J. Cai , D. W. Zhang , G. Zhao , Signal Transduct. Target. Ther. 2023, 8, 304. 37582956 10.1038/s41392-023-01503-7 PMC10427715

[71] L. V. Sinclair , T. Youdale , L. Spinelli , M. Gakovic , A. J. Langlands , S. Pathak , A. J. M. Howden , I. G. Ganley , D A. Cantrell , Nat. Immunol. 2025, 26, 429. 40016525 10.1038/s41590-025-02090-1 PMC11876071

[72] D. Denk , V. Petrocelli , C. Conche , M. Drachsler , P. K. Ziegler , A. Braun , A. Kress , A. M. Nicolas , K. Mohs , C. Becker , M. F. Neurath , H. F. Farin , C. J. Buchholz , P. A. Andreux , C. Rinsch , F R. Greten , Immunity 2022, 55, 2059. 36351375 10.1016/j.immuni.2022.09.014

[73] S. S. Gupta , R. Sharp , C. Hofferek , L. Kuai , G. W. Dorn 2nd , J. Wang , M. Chen , Cell Rep. 2019, 29, 1862. 31722203 10.1016/j.celrep.2019.10.032 PMC6886713

[74] K. M. Wragg , M. J. Worley , J. C. Deng , M. Salmon , D R. Goldstein , Am. J. Transplant 2024, 24, 2174. 39142471 10.1016/j.ajt.2024.08.005 PMC11588513

[75] H. Shuwen , W. Yinhang , Z. Jing , Y. Qiang , J. Yizhen , Q. Quan , J. Yin , L. Jiang , Y. Xi , Cancer Immunol. Immunother. 2023, 72, 4441. 37919522 10.1007/s00262-023-03555-8 PMC10991466

[76] A. Akhtar , M. Shakir , M. S. Ansari , F. M. I. Divya , V. Chauhan , A. Singh , R. Alam , I. Azmi , S. Sharma , M. Pracha , I. M. Uddin , U. Bashir , S. N. Shahni , R. Chaudhuri , S. Albogami , R. Ganguly , S. Sagar , V. P. Singh , G. Kharya , A. K. Srivastava , U. Mabalirajan , S. S. Roy , I. Rahman , T. Ahmad , Cell Rep. Med. 2025, 6, 102021. 40107240 10.1016/j.xcrm.2025.102021 PMC11970383

[77] H. Zhang , J. Liu , W. Yuan , Q. Zhang , X. Luo , Y. Li , Y. Peng , J. Feng , X. Liu , J. Chen , Y. Zhou , J. Lv , N. Zhou , J. Ma , K. Tang , B. Huang , Nat. Cell Biol. 2024, 26, 1892. 39261719 10.1038/s41556-024-01503-x

[78] L. Vaillant , W. Akhter , J. Nakhle , M. Simon , M. Villalba , C. Jorgensen , M. L. Vignais , J. Hernandez , Stem. Cell Res. Ther. 2024, 15, 394. 39497203 10.1186/s13287-024-03980-1 PMC11536934

[79] J. G. Baldwin , C. Heuser‐Loy , T. Saha , R. C. Schelker , D. Slavkovic‐Lukic , N. Strieder , I. Hernandez‐Lopez , N. Rana , M. Barden , F. Mastrogiovanni , A. Martín‐Santos , A. Raimondi , P. Brohawn , B. W. Higgs , C. Gebhard , V. Kapoor , W. G. Telford , S. Gautam , M. Xydia , P. Beckhove , S. Frischholz , K. Schober , Z. Kontarakis , J. E. Corn , M. Iannacone , D. Inverso , M. Rehli , J. Fioravanti , S. Sengupta , L. Gattinoni , Cell 2024, 187, 6614. 39276774 10.1016/j.cell.2024.08.029 PMC11623344

[80] T. Saha , C. Dash , R. Jayabalan , S. Khiste , A. Kulkarni , K. Kurmi , J. Mondal , P. K. Majumder , A. Bardia , H. L. Jang , S. Sengupta , Nat. Nanotechnol. 2022, 17, 98. 34795441 10.1038/s41565-021-01000-4 PMC10071558

[81] A.‐R. Pg , Cell Metab. 2016, 23, 967. 27304497 10.1016/j.cmet.2016.05.018

[82] M. Lisci , G M. Griffiths , Trends Cell Biol. 2023, 33, 138. 35753961 10.1016/j.tcb.2022.05.007

[83] Q. Du , N. Ning , X. Zhao , F. Liu , S. Zhang , Y. Xia , F. Li , S. Yuan , X. Xie , M. Zhu , Z. Huang , Z. Tang , J. Wang , R. He , X P. Yang , Theranostics 2025, 15, 1304. 39816692 10.7150/thno.101298 PMC11729555

[84] X. Wang , H. Zhang , Y. Wang , L. Bramasole , K. Guo , F. Mourtada , T. Meul , Q. Hu , V. Viteri , I. Kammerl , M. Konigshoff , M. Lehmann , T. Magg , F. Hauck , I. E. Fernandez , EMBO J. 2023, 42, 110597. 10.15252/embj.2022110597 PMC10106989 36912165

[85] M. Lisci , P. R. Barton , L. O. Randzavola , C. Y. Ma , J. M. Marchingo , D. A. Cantrell , V. Paupe , J. Prudent , J. C. Stinchcombe , G M. Griffiths , Science 2021, 374, 9977. 10.1126/science.abe9977 34648346

[86] A. N. Chen , Y. Luo , Y. H. Yang , J. T. Fu , X. M. Geng , J. P. Shi , Front. Immunol. 2021, 12, 688910. 34177945 10.3389/fimmu.2021.688910 PMC8222712

[87] Y. Mao , J. Zhang , Q. Zhou , X. He , Z. Zheng , Y. Wei , K. Zhou , Y. Lin , H. Yu , H. Zhang , Y. Zhou , P. Lin , B. Wu , Y. Yuan , J. Zhao , W. Xu , S. Zhao , Cell Res. 2024, 34, 13. 38163844 10.1038/s41422-023-00864-6 PMC10770133

[88] W. Weng , Z. He , Z. Ma , J. Huang , Y. Han , Q. Feng , W. Qi , Y. Peng , J. Wang , J. Gu , W. Wang , Y. Lin , G. Jiang , J. Jiang , J. Feng , Cell Death Differ. 2025, 32, 530. 39496783 10.1038/s41418-024-01408-0 PMC11894137

[89] W. Hong , X. Zeng , H. Wang , X. Tan , Y. Tian , H. Hu , M. Ashrafizadeh , G. Sethi , H. Huang , C. Duan , Pharmacol. Res. 2024, 205, 107228. 38810904 10.1016/j.phrs.2024.107228

[90] H. She , Y. Hu , G. Zhao , Y. Du , Y. Wu , W. Chen , Y. Li , Y. Wang , L. Tan , Y. Zhou , J. Zheng , Q. Li , H. Yan , Q. Mao , D. Zuo , L. Liu , T. Li , Adv. Sci. 2024, 11, 2409499. 10.1002/advs.202409499 PMC11672254 39467114

[91] M. Abd Hamid , Y. Peng , T. Dong , Cell Mol. Immunol. 2020, 17, 684. 32451453 10.1038/s41423-020-0468-x PMC7331575

[92] E. Zacharioudakis , B. Agianian , K Mv V. , N. Biris , T. P. Garner , I. Rabinovich‐Nikitin , A. T. Ouchida , V. Margulets , L. U. Nordstrøm , J. S. Riley , I. Dolgalev , Y. Chen , A. J. H. Wittig , R. Pekson , C. Mathew , P. Wei , A. Tsirigos , S. W. G. Tait , L. A. Kirshenbaum , R. N. Kitsis , E. Gavathiotis , Nat. Commun. 2022, 13, 3775. 35798717 10.1038/s41467-022-31324-1 PMC9262907

[93] C. B. Nava Lauson , S. Tiberti , P. A. Corsetto , F. Conte , P. Tyagi , M. Machwirth , S. Ebert , A. Loffreda , L. Scheller , D. Sheta , Z. Mokhtari , T. Peters , A. T. Raman , F. Greco , A. M. Rizzo , A. Beilhack , G. Signore , N. Tumino , P. Vacca , L. A. McDonnell , A. Raimondi , P. D. Greenberg , J. B. Huppa , S. Cardaci , I. Caruana , S. Rodighiero , L. Nezi , T. Manzo , Cell Metab. 2023, 35, 633. 36898381 10.1016/j.cmet.2023.02.013

[94] H. Wan , B. Xu , N. Zhu , B. Ren , Tumori 2020, 106, 55. 31451071 10.1177/0300891619868287

[95] P. S. Chowdhury , K. Chamoto , A. Kumar , T. Honjo , Cancer Immunol. Res. 2018, 6, 1375. 30143538 10.1158/2326-6066.CIR-18-0095

[96] H. J. Choi , S. Y. Jang , E S. Hwang , Mol. Cells 2015, 38, 918. 26442863 10.14348/molcells.2015.0168 PMC4625074

[97] Y. Xiao , N. Pang , S. Ma , M. Gao , L. Yang , Nutrients 2024, 16, 3577. 39519411 10.3390/nu16213577 PMC11547570

[98] I. Montali , C. Ceccatelli Berti , M. Morselli , G. Acerbi , V. Barili , G. Pedrazzi , B. Montanini , C. Boni , A. Alfieri , M. Pesci , A. Loglio , E. Degasperi , M. Borghi , R. Perbellini , A. Penna , D. Laccabue , M. Rossi , A. Vecchi , C. Tiezzi , V. Reverberi , C. Boarini , G. Abbati , M. Massari , P. Lampertico , G. Missale , C. Ferrari , P. Fisicaro , J. Hepatol. 2023, 79, 50. 36893853 10.1016/j.jhep.2023.02.035

[99] M. Al‐Habsi , K. Chamoto , K. Matsumoto , N. Nomura , B. Zhang , Y. Sugiura , K. Sonomura , A. Maharani , Y. Nakajima , Y. Wu , Y. Nomura , R. Menzies , M. Tajima , K. Kitaoka , Y. Haku , S. Delghandi , K. Yurimoto , F. Matsuda , S. Iwata , T. Ogura , S. Fagarasan , T. Honjo , Science 2022, 378, 3510. 10.1126/science.abj3510 36302005

[100] J. Ma , L. Tang , Y. Tan , J. Xiao , K. Wei , X. Zhang , Y. Ma , S. Tong , J. Chen , N. Zhou , L. Yang , Z. Lei , Y. Li , J. Lv , J. Liu , H. Zhang , K. Tang , Y. Zhang , B. Huang , Nat. Immunol. 2024, 25, 552. 38263463 10.1038/s41590-023-01738-0 PMC10907288

[101] H. Wang , L. Wei , D. Mao , X. Che , X. Ye , Y. Liu , Y. Chen , Int. Immunopharmacol. 2023, 125, 111026. 37866315 10.1016/j.intimp.2023.111026

[102] Y. Ma , G. Yan , J. Guo , F. Li , H. Zheng , C. Wang , Y. Chen , Y. Ye , H. Dai , Z. Qi , G. Zhuang , Front. Immunol. 2021, 12, 616074. 33732240 10.3389/fimmu.2021.616074 PMC7959711

[103] L. Tang , X. Wang , R. Zhao , X. Chen , F. Wang , S. Xia , Q. Xiao , Q. Zhao , S. Yang , S. Y. Tan , J. Ethnopharmacol. 2023, 308, 116276. 36806340 10.1016/j.jep.2023.116276

[104] M. Malinee , G. N. Pandian , H. Sugiyama , Cell Chem. Biol. 2022, 29, 463. 34520746 10.1016/j.chembiol.2021.08.001

[105] H. Guo , Y. Liu , X. Li , H. Wang , D. Mao , L. Wei , X. Ye , D. Qu , J. Huo , Y. Chen , ACS Nano 2023, 17, 23829 37991391 10.1021/acsnano.3c07885 PMC10722610

[106] D. O'Sullivan , M. A. Stanczak , M. Villa , F. M. Uhl , M. Corrado , R. I. Klein Geltink , D. E. Sanin , P. Apostolova , N. Rana , J. Edwards‐Hicks , K. M. Grzes , A. M. Kabat , R. L. Kyle , M. Fabri , J. D. Curtis , M. D. Buck , A. E. Patterson , A. Regina , C. S. Field , F. Baixauli , D. J. Puleston , E. J. Pearce , R. Zeiser , E. L. Pearce , Proc. Natl. Acad. Sci. U.S.A. 2021, 118. 10.1073/pnas.2023752118 PMC8237659 34161266

[107] J. W. Zhai , L. L. Lv , J. J. Wu , Y. X. Zhang , Y. Shen , Q. X. Qu , C. Chen , Immunol. Lett. 2023, 263, 61. 37805094 10.1016/j.imlet.2023.10.002

[108] M. H. Wu , F. Valenca‐Pereira , F. Cendali , E. L. Giddings , C. Pham‐Danis , M. C. Yarnell , A. J. Novak , T. M. Brunetti , S. B. Thompson , J. Henao‐Mejia , R. A. Flavell , A. D'Alessandro , M. E. Kohler , M. Rincon , Nat. Commun. 2024, 15, 4444. 38789421 10.1038/s41467-024-48653-y PMC11126743

[109] O. U. Kawalekar , R. S. O'Connor , J. A. Fraietta , L. Guo , S. E. McGettigan , A. D. Posey Jr. , P. R. Patel , S. Guedan , J. Scholler , B. Keith , N. W. Snyder , I. A. Blair , M. C. Milone , C. H. June , Immunity 2016, 44, 380. 26885860 10.1016/j.immuni.2016.01.021 PMC13498396

[110] M. Abdoli Shadbad , N. Hemmat , V. Khaze Shahgoli , A. Derakhshani , F. Baradaran , O. Brunetti , R. Fasano , R. Bernardini , N. Silvestris , B. Baradaran , Front. Immunol. 2021, 12, 788211. 35126356 10.3389/fimmu.2021.788211 PMC8807490

[111] H. Liang , W. Fu , W. Yu , Z. Cao , E. Liu , F. Sun , X. Kong , Y. Gao , Y. Zhou , Front. Immunol. 2022, 13, 1061448. 36420255 10.3389/fimmu.2022.1061448 PMC9676649

[112] L. Lin , R. Ren , Q. Xiong , C. Zheng , B. Yang , H. Wang , Autoimmun. Rev. 2024, 23, 103583. 39084278 10.1016/j.autrev.2024.103583

[113] C. M. Jackson , A. Pant , W. Dinalankara , J. Choi , A. Jain , R. Nitta , E. Yazigi , L. Saleh , L. Zhao , T. R. Nirschl , C. M. Kochel , B. Hwa‐Lin Bergsneider , D. Routkevitch , K. Patel , K. B. Cho , S. Tzeng , S. Y. Neshat , Y. H. Kim , B. J. Smith , M. C. Ramello , E. Sotillo , X. Wang , J. J. Green , C. Bettegowda , G. Li , H. Brem , C. L. Mackall , D. M. Pardoll , C. G. Drake , L. Marchionni , et al., Immunity 2024, 57, 1864. 39111315 10.1016/j.immuni.2024.07.003 PMC11324406

[114] J. A. Godoy , J. A. Rios , P. Picón‐Pagès , V. Herrera‐Fernández , B. Swaby , G. Crepin , R. Vicente , J. M. Fernández‐Fernández , M FJ. Mitostasis , Biomolecules. 2021, 11. 10.3390/biom11071012 PMC8301949 34356637

